# Interference of Co-Amplified Nuclear Mitochondrial DNA Sequences on the Determination of Human mtDNA Heteroplasmy by Using the SURVEYOR Nuclease and the WAVE HS System

**DOI:** 10.1371/journal.pone.0092817

**Published:** 2014-03-24

**Authors:** Hsiu-Chuan Yen, Shiue-Li Li, Wei-Chien Hsu, Petrus Tang

**Affiliations:** 1 Department and Graduate Institute of Medical Biotechnology and Laboratory Sciences, College of Medicine, Chang Gung University, Tao-Yuan, Taiwan; 2 Department of Public Health and Parasitology, College of Medicine, Chang Gung University, Tao-Yuan, Taiwan; RIKEN Advanced Science Institute, Japan

## Abstract

High-sensitivity and high-throughput mutation detection techniques are useful for screening the homoplasmy or heteroplasmy status of mitochondrial DNA (mtDNA), but might be susceptible to interference from nuclear mitochondrial DNA sequences (NUMTs) co-amplified during polymerase chain reaction (PCR). In this study, we first evaluated the platform of SURVEYOR Nuclease digestion of heteroduplexed DNA followed by the detection of cleaved DNA by using the WAVE HS System (SN/WAVE-HS) for detecting human mtDNA variants and found that its performance was slightly better than that of denaturing high-performance liquid chromatography (DHPLC). The potential interference from co-amplified NUMTs on screening mtDNA heteroplasmy when using these 2 highly sensitive techniques was further examined by using 2 published primer sets containing a total of 65 primer pairs, which were originally designed to be used with one of the 2 techniques. We confirmed that 24 primer pairs could amplify NUMTs by conducting bioinformatic analysis and PCR with the DNA from 143B-ρ^0^ cells. Using mtDNA extracted from the mitochondria of human 143B cells and a cybrid line with the nuclear background of 143B-ρ^0^ cells, we demonstrated that NUMTs could affect the patterns of chromatograms for cell DNA during SN-WAVE/HS analysis of mtDNA, leading to incorrect judgment of mtDNA homoplasmy or heteroplasmy status. However, we observed such interference only in 2 of 24 primer pairs selected, and did not observe such effects during DHPLC analysis. These results indicate that NUMTs can affect the screening of low-level mtDNA variants, but it might not be predicted by bioinformatic analysis or the amplification of DNA from 143B-ρ^0^ cells. Therefore, using purified mtDNA from cultured cells with proven purity to evaluate the effects of NUMTs from a primer pair on mtDNA detection by using PCR-based high-sensitivity methods prior to the use of a primer pair in real studies would be a more practical strategy.

## Introduction

Human mitochondrial DNA (mtDNA) is a circular genome with 16569 base pairs (bp), and exists as hundreds to thousands of copies in the mitochondrial matrix. It encodes genes for 2 rRNA genes, 22 tRNA genes, and 13 mRNA genes for subunits of Complexes I, III, IV, and V in oxidative phosphorylation. Several genetic features of mtDNA differ from those of nuclear DNA (nDNA), such as maternal inheritance, replicative segregation during cell division, and high sequence evolution rate [1]. Pathogenic mutations of mtDNA transmitted maternally can cause genetic diseases, whereas inherited ancient adaptive variants may increase the risk of certain diseases [2]. Somatic mtDNA mutations in aging-related diseases and cancers in humans have also been investigated extensively [2], [3]. Because decision-making on homoplasmy or heteroplasmy status of mtDNA mutations can have implications, such as the determination of maternal inheritance or the differentiation between somatic mutations and genetic drift in cancer patients, detection of low-percentage heteroplasmy of mtDNA by high-sensitivity techniques, such as denaturing high-performance liquid chromatography (DHPLC) and pyrosequencing (PSQ), has become an increasing research interest, as we discussed previously [4]. PSQ is a powerful technique for the quantification of mtDNA heteroplasmy for a known single nucleotide polymorphism (SNP) or mutation without the need of establishing a standard curve [4], [5], whereas allele-refractory mutation system (AMRS)-based quantitative polymerase chain reaction (qPCR) represents a highly sensitive technique that requires a standard curve made by mixing 2 DNA samples containing different alleles [6]. However, these kinds of methods are not suitable for the search on unknown variants or mutations, which are often present at low-level heteroplasmy as well, over entire mtDNA in various studies. Generating a standard curve for any potential novel SNP or mutation detected by direct sequencing under such scenario is also not practical. In contrast, DHPLC represents a kind of sensitive and high-throughput, although qualitative, technique to screen heteroplasmic variants for entire mtDNA [7], [8].

The detection of mutation by using the CEL 1 endonuclease from celery, which exerts 2 independent cuts at 3′-side of mismatched nucleotides on 2 strands of heteroduplexed DNA without sequence specificity [9], has been commercialized as SURVEYOR Nuclease (SN) in the SURVEYOR Mutation Detection Kit. The platform of SN digestion followed by the detection of DNA fragments by using the WAVE HS System, designated as SN/WAVE-HS in this paper, is a high-sensitivity detection technique, in which an injector for the fluorescent dye and a fluorescent detector are equipped on the WAVE System used in traditional DHPLC analysis. Janne et al. has applied this platform for high-sensitivity mutation screening of the epidermal growth factor receptor (EGRF) gene in human cancer specimens [10], but it has not been applied for the detection of mtDNA mutation. Although Bannwarth et al. have developed 17 primer pairs for screening mutations of entire human mtDNA by using SN analysis, they detected cleaved DNA fragments by using agarose gel electrophoresis [11], [12]. Because SN/WAVE-HS analysis does not require the testing on optimal temperatures and can be used to analyze amplicons much longer than 500 bp, it is very likely to be a more superior technique than DHPLC for the efficient screening of unknown heteroplasmic variants or mutations in entire mtDNA.

The abbreviation NUMTs (or Numts) can refer to nuclear mtDNA sequences or mt DNA-like sequences in the nucleus [13], nuclear mitochondrial pseudogenes [14], or nuclear DNA sequences of mitochondrial origin [15]. Studies have indicated that NUMTs present in modern eukaryotes originated from the transfer and integration of ancient mtDNA fragments into nDNA at different stages during evolution and therefore have similar, but not identical, sequences to modern mtDNA sequences because of the much faster rate of mutation of mtDNA compared with nDNA [14]. According to results of bioinformatic analyses in different studies, human NUMTs, estimated to occupy 0.016% of nDNA [16], with the lengths ranging from 28 to 14 915 bp [13], exist in all human chromosomes as a single copy, multiple copies, or in highly rearranged forms, and match with all regions of human mtDNA sequences at various frequencies and with different similarities [13], [17], [18]. However, results from bioinformatic analysis of human NUMTs, such as lengths of NUMTs and location on chromosomes, varied among the different studies because of the use of different searching methods or matching parameters.

The possible misinterpretation of novel heteroplasmic mtDNA mutations in human diseases because of NUMTs [14], [19], [20] and the development of strategies to avoid interference from NUMTs during mtDNA detection [21], [22] have recently attracted considerable research attention. However, certain assumptions and approaches employed in previous studies require further examination. First, the disputation of the novelty of a reported mtDNA variant based on the results of database searches, Internet searches, and phylogenetic analyses might not necessarily indicate that the variant was indeed from NUMTs unless otherwise proven by solid experimental data. Because the copy number of the mtDNA outnumbers that of NUMTs on nDNA in regular DNA samples and direct sequencing has poor sensitivity for the detection of heteroplasmy, matching heteroplasmic variants from sequencing results to predicted NUMTs by using bioinformatics approaches might not disprove the presence of actual mutations. Second, because the same amount of DNA from ρ^0^ cells has much higher absolute amount of nDNA than the nDNA in DNA samples from regular cells containing both nDNA and mtDNA, the amplification of NUMTs from the DNA of ρ^0^ cells by a primer pair should not automatically infer the interference of NUMTs on mtDNA detection by any technique using this primer pair. Third, because some regions of mtDNA display high similarity to predicted NUMTs, the design of a usable primer pair specific to mtDNA might be problematic if low matching with NUMTs databases and inability to amplify the DNA of ρ^0^ cells are the prerequisites. Finally, the use of pure mtDNA without nDNA contamination should be able to prove or disprove the possible interference of NUMTs on mtDNA detection by using a PCR-based technique. However, it is essential to demonstrate the purity of such mtDNA fractions through well-controlled PCR examinations because any nDNA contamination detectable by the PCR is likely to be detected by high-sensitivity techniques.

In this study, we first established our SN/WAVE-HS methods for mtDNA detection, and compared the performance of SN/WAVE-HS analysis with DHPLC analysis for the detection of different percentages of SNP by using 2 cloned plasmid DNA samples containing mtDNA fragments from human blood DNA. We then examined possible interference from NUMTs on the interpretation of heteroplasmy or homoplasmy during mtDNA detection by the DHPLC and SN/WAVE-HS techniques. Two published primer sets originally designed for the screening of unknown mutations of entire human mtDNA were selected for the evaluations. The primer set designed by Meierhofer et al., containing 48 primer pairs, was originally used for DHPLC analysis [7], whereas the 17 primer pairs designed by Bannwarth et al. were used in the SN analysis, followed by agarose gel detection [11]. These 2 primer sets were designated as the DM primer set and SB primer set, respectively, in this study according to the names of the first authors in these two publications.

## Materials and Methods

### Ethics Statement

The parts of this study that involved the use of human blood samples from 2 healthy human volunteers and the analysis of mtDNA sequences for the blood DNA samples from these 2 subjects were conducted with approval from the Institutional Review Boards of Chang Gung Memorial Hospital in accordance with the Declaration of Helsinki for Human Research. The blood samples were obtained with written informed consent.

### Cell Lines and Culture of Cells

143B cells, an osteosarcoma cell line (ATCC CRL 8303), 143B-ρ^0^ cells (clone 87 derived from 143B cells), and a cybrid cell line were obtained from Professor Douglas Wallace. The cybrid cells line was generated by fusion of the 143B-ρ^0^ cells and the cytoplast from patients, followed by further selection of a wild-type cybrid clone as a control cybrid of the cybrid containing a pathogenic mtDNA mutation [23]. 143B cells and the cybrid cells were grown in Dulbecco's modified Eagle's medium (DMEM) containing 10% fetal bovine serum, 4.5 g/L glucose, and 110 μg/mL pyruvate. 143B-ρ^0^ cells were cultured in the same medium with additional supplementation of 50 μg/mL uridine [24]. The growth of all cell lines was maintained under the conditions of 5% CO_2_ and 37°C in a CO_2_ incubator.

### DNA Isolation from Whole Blood and Cultured Cells

For isolation of DNA from whole blood and cultured cells, the Gentra Puregene Blood Kit and Gentra Puregene Cell Kit (QIAGEN) were used, respectively, and the procedures described in the Genetra Puregene Handbook (third edition) from QIAGEN were followed. The DNA obtained by this way contained both nDNA and mtDNA and so was referred as total DNA in this paper.

### Isolation of Mitochondria from Cultured Cells for Isolation of mtDNA and the Verification on the Purity of Isolated mtDNA

The procedures of mitochondrial isolation were modified from our previous protocols for Western blot analysis [25] with additional centrifugation steps on supernatants after the first 1000×*g* centrifugation of the homogenate and the DNase I treatment step for the mitochondrial fraction because PCR is a much more sensitive detection method than Western blot analysis. In brief, cells on dishes were washed by phosphate-buffered saline (PBS) and then scraped with the mitochondrial isolation solution previously described [25]. Cell pellet collected from centrifugation at 1000×*g* was homogenized in the mitochondrial isolation solution followed by centrifugation at 1000×*g* for 10 min at 4°C. The first supernatant was further centrifuged at 2000×*g*, followed by centrifugation at 3000×*g* for the second supernatant. The final supernatant was centrifuged at 10000×*g* for 15 min at 4°C. The pellet or the isolated mitochondrial fraction was washed and resuspended in the mitochondrial isolation solution. DNase I solution was added to this mitochondrial suspension to a final concentration of 10 μg/mL and incubated at 37°C for 5 min to remove any nDNA attached to the surface of isolated mitochondria [26]. The mitochondrial suspension was centrifuged and the resulting pellet was washed with the mitochondrial isolation solution, followed by the mixing of the pellet with the Cell Lysis Solution from Gentra Puregene Cell Kit. The same DNA isolation protocols used for cultured cells were then carried out to extract mtDNA from isolated mitochondria, but the Glycogen Solution from the kit was added into isopropanol for the DNA precipitation step.

To verify the purity of isolated mtDNA, we compared the results of PCR for mtDNA from mitochondria of 143B cells, total DNA from 143B cells containing both nDNA and mtDNA, and DNA from 143B-ρ^0^ cells containing only nDNA by using nDNA-specific primers and mtDNA-specific primers. The mtDNA-specific primers that did not amplify DNA from 143B-ρ^0^ cells for the amplicon of nt 3374–3894 have been previously described [4]. The forward primer 5′-GAAACTTAGATCCAGGTGTCGC-3′ and reverse primers 5′-AGCAGCAATTTGTAAGTGTCCC-3′ were designed as nDNA-specific primers, which amplified a fragment of human *SOD2* gene across an exon and an intron with the length of 293 bp.

### Automatic Direct Sequencing and Sequence Analysis

For blood DNA from 2 healthy volunteers (subject A and subject B), entire mtDNA was sequenced to obtain information on nucleotide positions with SNPs between these 2 subjects. The primers from MitoSEQr Resequencing System (ABI) were used to generate 46 amplicons of human mtDNA by PCR according to the manufacturer's instruction of the AmpliTag Gold PCR Master Mix (ABI). On the other hand, when there were obvious heteroduplex signals during DHPLC analysis, the PCR products of the DM or SB amplicons from 143B-ρ^0^ cells, 143B cells, and the cybrid cells were sequenced by using the DM or SB primer pairs. The PCR products were sent to Genomic Inc. (Taiwan) for automatic direct sequencing, which was carried out by the ABI 3730XL System. Sequencing results were compared with the revised Cambridge reference sequence/rCRS (GenBank ID: NC_012920) by using the SeqScape software version 2.5 (ABI).

### Cloning of mtDNA Fragments from Blood DNA and Subsequent Plasmid Purification

Based on the sequencing results, we found that there was only one SNP at nt 12705 in the amplicon of nt 12566-12895 from mtDNA, in which subject A and B had T allele and C allele, respectively. This fragment with the size of 330 bp from subject A and B was amplified by PCR by using the forward primer 5′-CCCTAAGCTTCAAACTAGACTAC-3′ and reverse primer 5′-CTAAGGCGAGGATGAAACCGATA and the Taq DNA polymerase (Invitrogen). The PCR products were cloned by using the T&A Cloning Vector Kit and the ECOS competent cells (Yeastern Biotech, Taiwan) according to the manufacturer's instruction. Two sequence-verified colonies with T and C allele were amplified. Plasmids purified by the GENO Mini Plasmid Kit (Genomics BioScience and Technology, Taiwan) from these two clones for allele T and C allele were designated as plasmid A and plasmid B, respectively.

### Bioinformatic Analysis to Evaluate the Matching Conditions of Primers with NUMTs

As previously described in our publication [4], to examine how primers designed for human mtDNA matched with NUMTs, we routinely conducted searches by using the Basic Local Alignment Search Tool (BLAST) not only against the nucleotide database in National Center for Biotechnology Information (NCBI), but also against a local database containing the rCRS and the 228 accession numbers currently available on GenBank from the 247 accession numbers that were identified to contain NUMTs sequences by Mishmar et al. [13], which were also available on the MITOMAP (http://www.mitomap.org/MITOMAP/PseudogeneList). In this study, we used the BioEdit online tool (http://www.mbio.ncsu.edu/bioedit/bioedit.html) to create the local database. The default settings for the algorithm parameters were used for the BLAST searches on either NCBI or BioEdit. This BLAST searches were used in this study to examine whether any primer pair from DM and SB primer sets had high probability to amplify NUMTs. When there were matching between a primer and nDNA sequences, the results were categorized into 3 matching conditions: perfect match, one mismatch, and two or more mismatches. When there was one perfect match or only one mismatch, the E values from the BLAST searches were all below 0.3. Moreover, we also examined whether a primer pair matched to the same contig on the same chromosome. Subsequently, the nucleotide locations and lengths of the marched regions matched by a specific primer pair could be known.

### PCR Reactions for DHPLC and SN/WAVE-HS Analyses

Optimase DNA polymerase (Transgenomic) and the GeneAmp PCR system 9800 (Applied Biosystems) were used for all PCR reactions. For the 12566–12895 amplicon, the PCR mixture in 30 μL contained the reaction buffer, 1.5 mM MgSO_4_, 0.4 μM primers, 0.2 mM dNTP, 0.75 U polymerase, and 0.03 ng plasmid DNA or 6 ng blood DNA. The PCR conditions for this amplicon followed the suggestion described in the manual for the SN kit except the use of 35 cycles and 64°C as the annealing temperature. When using DM primers, PCR was carried out according to the conditions described by Meierhofer et al., in which one condition was applied for all amplicons [7], except the use of 36 ng DNA per 30 μL reaction. For SB primers, the same PCR conditions described by Bannwarth et al. [11] were used for all amplicons, in which 30 ng DNA was added per 30 μL reaction.

### DHPLC Analysis

The PCR product amplified from each DNA sample alone was subjected to heating-and-annealing reaction by using the PE2400 PCR machine (Perkin Elmer) for the generation of homoduplexes and heteroduplexes. The samples were first heated to 95°C for 4 min, and then cooled to 25°C at the rate of 1°C/min. Samples were then injected into the WAVE 3500A System equipped with the DNASep cartridge (Transgenomic) for repeated analysis of each sample at different column temperatures. For the mobile phase, buffer A consisted of 0.1 M trietylammonium acetate (TEAA), pH 7.0, whereas buffer B contained 25% acetonitrile in the TEAA buffer. The gradient profile for each amplicon was determined by the Navigator software of the WAVE System. The observation of 2 or more peaks on the chromatograms indicated the presence of both heteroduplexes and homoduplexes.

### SN/WAVE-HS Analysis

Heat-annealed PCR product in the amount of 6 μL was subjected to SN digestion by using the Transgenomic SURVEYOR Plus Mutation Detection Kit for Wave and Wave HS Systems for most of samples according to the instruction of the manufacturer except the use of half amount of Enhancer W2 and the Nuclease W. For the purpose of comparison, the Transgenomic SURVEYOR Mutation Detection Kit, a previous version of the kit that has been used in the literature, was also tested to perform SN digestion of plasmid DNA, for which 1 μL of Enhancer W and 1 μL of Nuclease W were mixed with 6 μL of DNA. When making uncut controls, the same DNA samples were mixed with all components except the Enhancer and Nuclease, which were replaced by water, and subjected to the same reaction conditions. DNA samples before (uncut groups) and after SN digestion (cut groups) were analyzed simultaneously by the ultraviolet (UV) detector (WAVE System) and the fluorescent detector (WAVE HS System), which was coupled with a device to mix the WAVE HS Staining Solution I (Transgenomic) with DNA samples before injection, at the column temperature of 50°C under the mode of Double-strand (DS) Multiple Fragments. Therefore, the separation of DNA was based on the size of dsDNA under a non-denaturing temperature. The chromatographic setting was designed according to the instruction in the SURVEYOR Plus Mutation Detection Kit when handling amplicons from DM primers, which was based on the amplicon size, but the minimum and maximum lengths for the analysis were set as 100 bp and 1050 bp, respectively, for amplicons from SB primers to shorten the run time. A 100-bp DNA ladder (New England Biolabs) was run in parallel with samples for each analysis under different chromatographic conditions. The identification of heteroduplexed DNA was determined by the observation of additional 2 or more peaks in the chromatograms from a SN-digested sample compared with that from the corresponding uncut control.

## Results

### Comparison of the Performance of SN/WAVE-HS and DHPLC for the Detection of mtDNA Heteroplasmy by Using Plasmid DNA and Blood DNA

**Figure 1:**
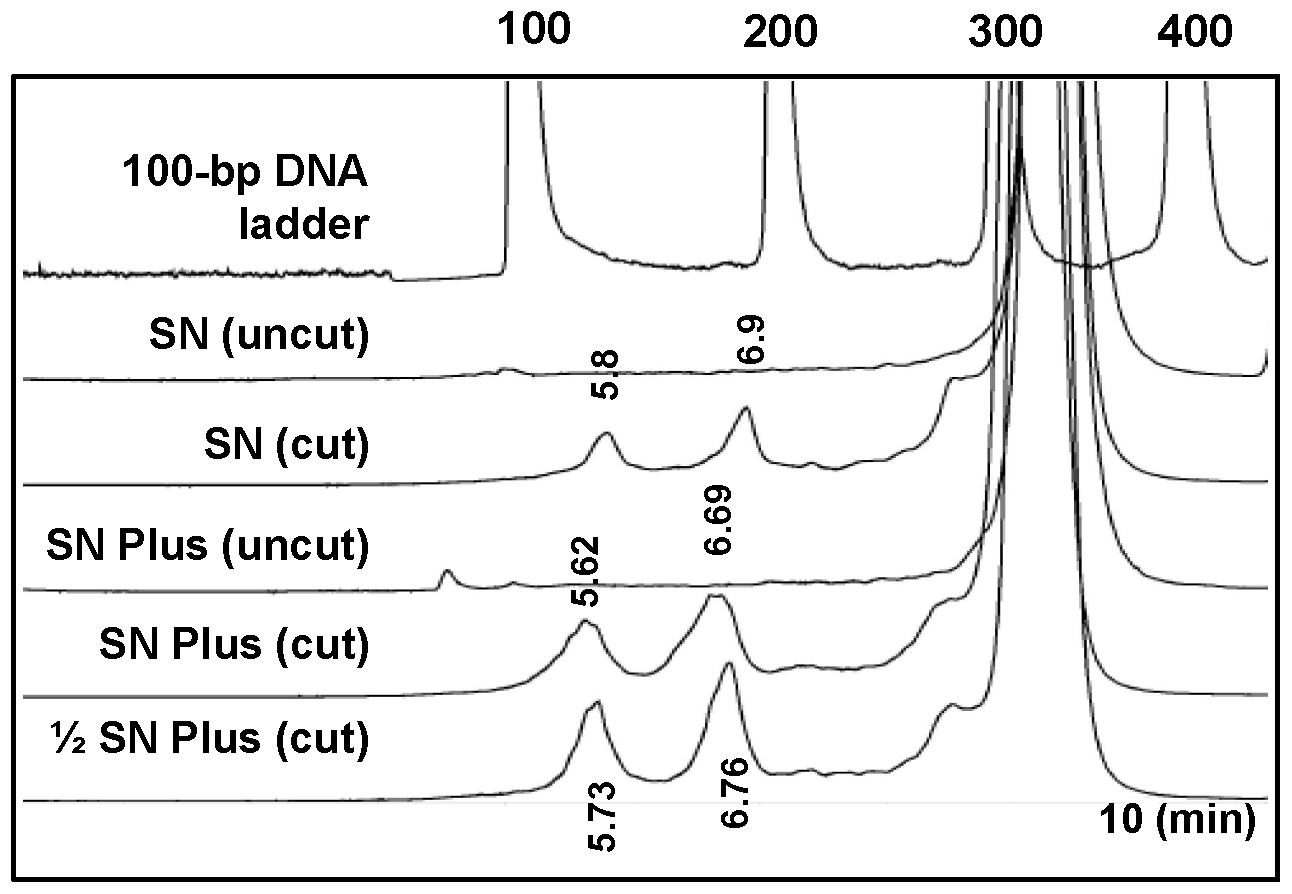
Comparison of the SURVEYOR Mutation Detection Kit and the SURVEYOR Plus Mutation Kit. A mixture of 50% plasmid A and 50% plasmid B was used as the template for the PCR. The expected size of the PCR product was 330 bp, whereas that of the two DNA fragments after SN cleavage was 130 bp and 190 bp. Results for the DNA samples without (uncut) and with (cut) SN digestion were compared. SN and SN Plus indicate the results obtained using the SURVEYOR Mutation Detection Kit and the SURVEYOR Plus Mutation Kit, respectively. 1/2 SN Plus refers to the use of 50% of the manufacturer's recommended amount of Enhancer W2 and Nuclease W when using the new kit. The numbers above the peaks of the chromatograms for the DNA samples indicate the retention times of the cleaved fragments. The numbers above the peaks of the DNA ladder indicate the sizes of the DNA markers. The large peak at the retention time after that of the 300-bp DNA marker indicates the uncleaved DNA.

So far SN analysis appeared in the literature all utilized the SURVEYOR Mutation Detection Kit (old SN kit) from Transgenomic, but only the new version of kit containing a new Enhancer formula and additional cofactors, SURVEYOR Plus Mutation Detection Kit (new SN Plus kit), was available since the year of 2010. We therefore compared the results from the 2 versions of the kits by using the template containing equal amount of plasmid A and plasmid B (50% plasmid B). After SN digestion, theoretically two fragments in the lengths of 139 bp and 190 bp should be generated and detected by SN/WAVE-HS. As shown in Figure 1, although we observed that SN digestion performed by the new kit, according to the manufacturer's instructions, produced higher signals for cleaved products when detected by the WAVE HS System, the new kit led to the generation of broader peaks, or peaks with shifted retention time, compared with those generated by the old kit. This finding was reproducible in another 2 independent experiments. We also observed the same phenomenon when using a mixture of Control G and Control C plasmids provided in the kit (data not shown). These results indicated substantial effects of non-specific digestion by SN when using the new kit. To resolve such problems without compromising the intensities of signals, we used 50% of the manufacturer's recommended amounts of Enhancer W2 and Nuclease W (Figure 1).

**Figure 2:**
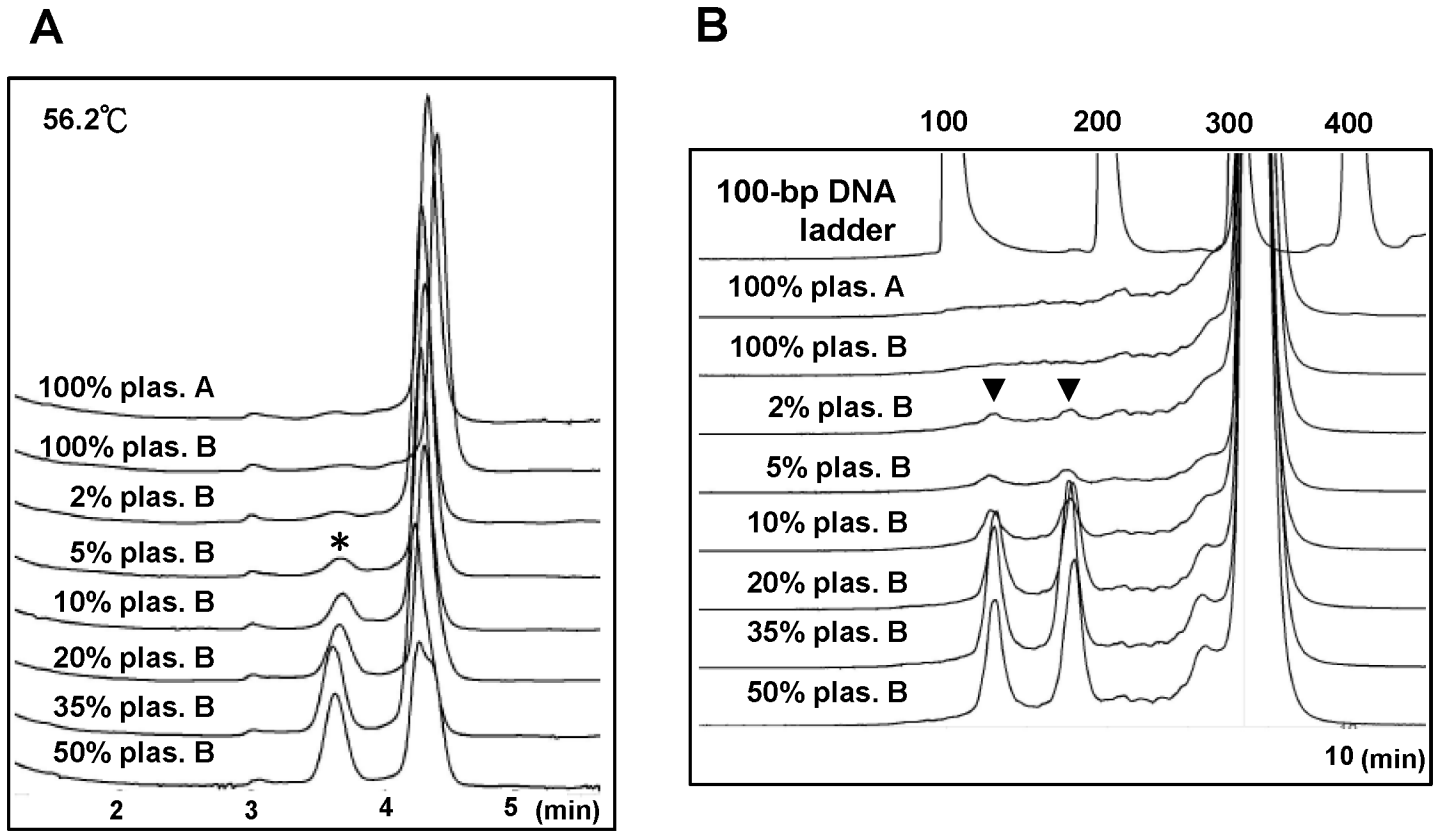
Evaluation of the detection of low-percentage variants on plasmids by using (A) DHPLC and (B) SN/WAVE-HS. The SURVEYOR Plus Mutation Kit was used for SN/WAVE-HS analysis. Templates with different percentages of plasmid B (indicated as plas. B) were prepared by mixing plasmid B with plasmid A. The appearance of at least 2 adjacent peaks on the chromatograms from DHPLC analysis indicated the presence of heteroduplexes (front peak) and homoduplexes (back peak) in the heat-annealed PCR products. The front heteroduplexes are indicated by the symbol of *. Two symbols of ▾ indicate the expected 139-bp and 190-bp DNA fragments after SN digestion of heat-annealed PCR products. The numbers above the peaks of the DNA ladder indicate the sizes of the DNA markers. The small peaks at 3 min were caused by noise from the system, which appeared in all chromatograms in the DHPLC analysis; therefore, were disregarded.

**Figure 3:**
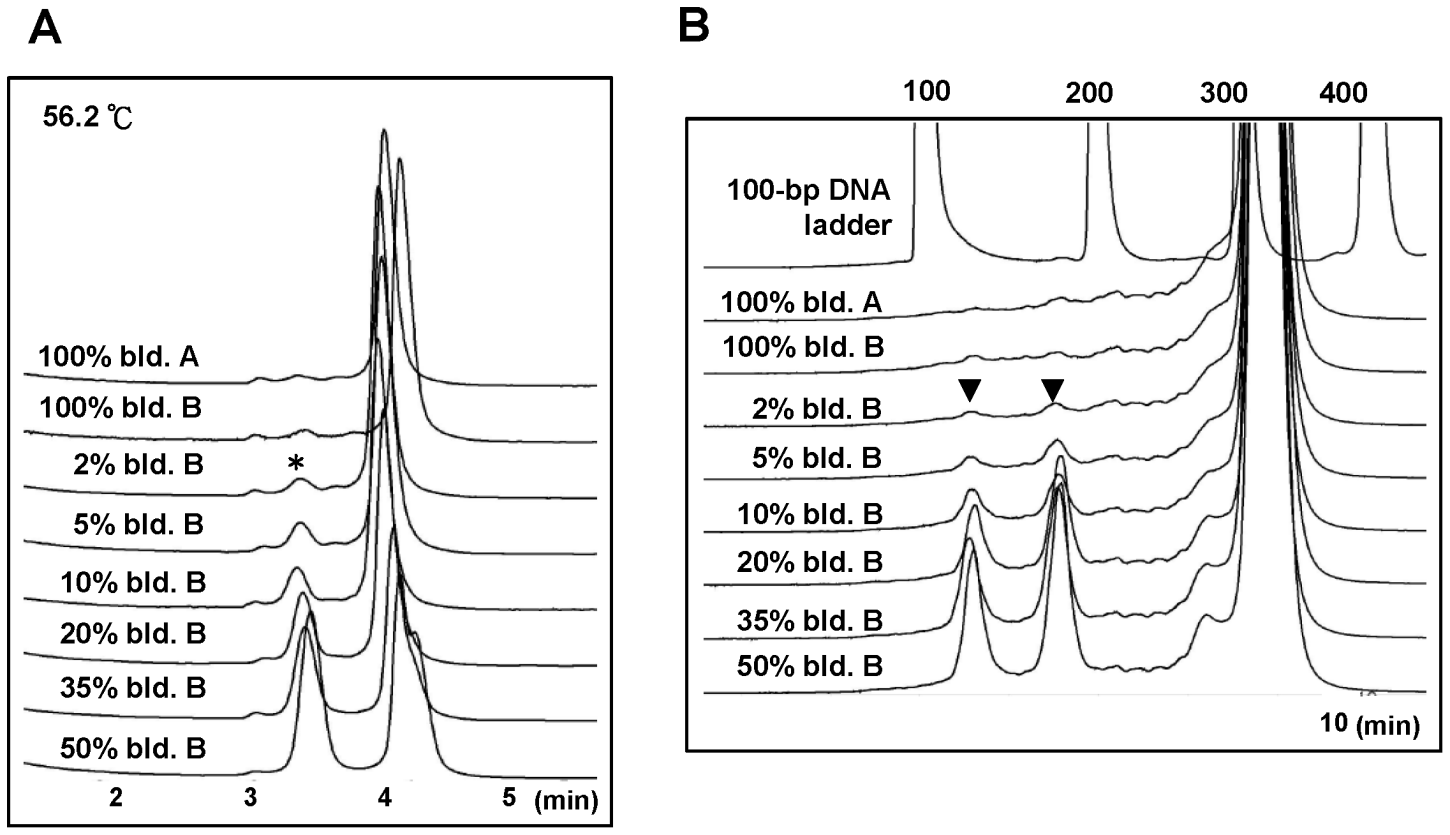
Evaluation of the detection of low-percentage variants on human blood DNA by using (A) DHPLC (B) and SN/WAVE-HS. The SURVEYOR Plus Mutation Kit was used for SN/WAVE-HS. Templates with different percentages of the DNA from blood B (designated as bld. B) were prepared by mixing the DNA from blood B with the DNA from blood A. The front heteroduplex peaks detected by DHPLC are indicated by *. ▾ indicates the expected 39-bp and 190-bp DNA fragments after SN digestion. The numbers above the peaks of the DNA ladder indicated the sizes of the DNA markers.

To compare the sensitivities of DHPLC and SN/WAVE-HS analyses for the detection of mtDNA heteroplasmy, we first tested the nt 12566–12895 amplicon with the SNP at position 12705 by mixing plasmid A and plasmid B to generate templates containing 2%–50% of plasmid B. This process excluded any possible interference from low-percentage of heteroplasmy undetectable by sequencing in blood DNA. The results from DHPLC analysis showed that the peaks of the heteroduplexes could not be differentiated from the traces of 100% plasmid A or B until at least 5% of plasmid B remained in the template for the PCR (Figure 2A). In contrast, peaks for 2 cleaved products detected by SN/WAVE-HS were evident, although small, for the sample containing 2% plasmid B, in comparison with the traces of 100% plasmid A or B (Figure 2B). This finding indicated that the sensitivity of this platform was relatively, although marginally, higher than that of DHPLC. As expected, the peaks of the cleaved products simultaneously detected by the UV detector on the WAVE System were considerably less obvious than those detected by the fluorescent detector for the same DNA sample shown in Figure 2B because the cleaved products for the 10% plasmid B were barely detectable (Figure S1). The traces from the 2% plasmid B could not be differentiated from those from pure plasmid A or plasmid B when using the old version of SN kit (Figure S2), indicating that the relative sensitivity of the old kit was slightly lower than that of the new kit. We then applied the comparison shown in Figure 2 to samples containing blood DNA from subject A and subject B, designated as blood A and blood B, respectively, in different percentages. When comparing the chromatograms for blood samples containing different percentages of blood B with those for 100% blood A or 100% blood B, the peaks of heteroduplexes detected by DHPLC (Figure 3A) and cleaved fragments detected by SN/WAVE-HS (Figure 3B) were noticeable when the percentage of blood B was ≧ 2%. However, 100% blood A or 100% blood B (particularly blood B), appeared to exhibit a low percentage of heteroplasmy, despite sequencing results indicating homoplasmic status. Therefore, comparisons of sensitively could be problematic when using blood DNA.

### Evaluation of the Potential of the DM Primer Set for Amplifying NUMTs by Bioinformatic Analysis and PCR Using the DNA of 143B-ρ°Cells

**Figure 4:**
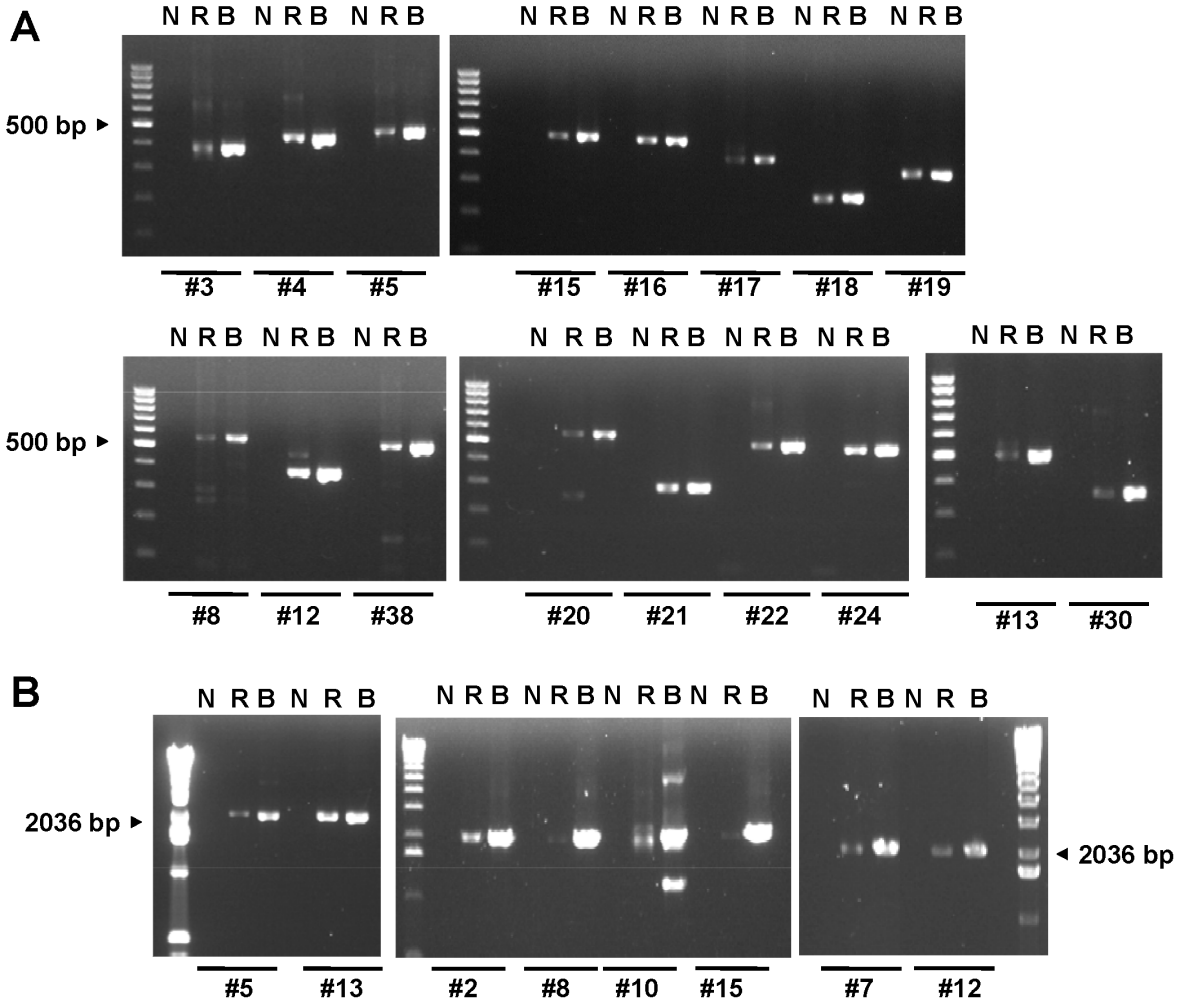
PCR products amplified from the DNA of 143B and 143B-ρ^0^ cells by using the selected primer pairs. (A) The 17 primer pairs selected from the DM primer set. (B) The 8 primer pairs selected from the SB primer set. A 100-bp DNA ladder and a 1-kb DNA ladder were used as the DNA sizing markers for the testing of the DM primer pairs and SB primer pairs, respectively. N, no-template control; R, DNA of 143B-ρ^0^ cells as the template; B, DNA of 143B cells as the template.

To evaluate whether any primer pair among the DM primer set had high potential to amplify NUMTs, we first performed a BLAST search for these primers against the NCBI nucleotide database and our local database created by BioEdit. We identified 23 primer pairs among the 48 primer pairs with high possibility of matching the sequences of accession numbers from the same chromosome under different matching conditions (Table 1). Notably, 14 primers pairs had perfect matches with the sequences of the accession numbers in one of the databases. Table 1 displays the accession numbers of matched nDNA, positions of the accession numbers matched by the primers, the sizes of matched NUMTs, and information on the regions of mtDNA to be amplified. These primer pairs matched with the sequences on chromosomes 1, 3, 5, 6, 11, 17, and X, with highest frequency for chromosomes 1, 5, and 6. The lengths of the highly matched NUMTs were the same as those of the targeted amplicons on mtDNA. To simplify our testing procedures, we then selected 17 primer pairs that had at least one primer that matched perfectly with sequences of at least one accession number of nDNA to further confirm the amplification of NUMTs by using the DNA of 143B-ρ^0^ cells. The amplicons tested were DM#3, DM#4, DM#5, DM#8, DM#12, DM#13, DM#15, DM#16, DM#17, DM#18, DM#19, DM#20, DM#21, DM#22, DM#24, DM#30, and DM#38. These primer pairs, except those for DM#13, DM1#17, and DM#30, exhibited perfect matches with sequences of at least one accession number for the same chromosome (Table 1). The results from agarose gel electrophoresis showed that these selected DM primers could produce visible PCR products from the DNA of 143B-ρ^0^ cells, although the yield from 143B-ρ^0^ cells was less than that from 143B cells, confirming that all the primers selected by our bioinformatic approach could all amplify NUMTs (Figure 4A). The PCR products from 143B-ρ^0^ cells with different sizes could be identified from the gel for several amplicons, such as DM#8, DM#12, and DM#20, although the sizes of major products appeared to be similar to those of targeted mtDNA sequences. However, these bands, which occurred in different sizes, were not present in the lanes for total DNA from 143B cells. Because we observed some nonspecific products from DM#8 primers, although in low quantities, we later excluded this amplicon for further testing as obtaining single band should be a common practice before further conducting mtDNA detection with the DHPLC or SN/WAVE-HS analysis.

**Table 1:** Results for the bioinformatics analysis on the matching of DM primers to NUMTs.

Amp. #	Primer sequences and the number of perfect matches with nDNA (Hits on NCBI, Hits on BioEdit)		Amplified mtDNA region (size)	NUMT location for the primer pair that matches to the same chromosome			
				Match	Chr. #	Accession #	Position (size)
**#1**	F	AGCACCCTATGTCGCAGTATC (0, 0)	108–638 (531 bp)	—	—	—	—
	R	GGTGATGTGAGCCCGTCTAAAC (2, 1)					
**#2**	F	CCAACCAAACCCCAAAGAC (4, 2)	548–964 (417 bp)	—	—	—	—
	R	GGGAGGGGGTGATCTAAAAC (0, 0)					
**#3**	F	GCCCCGCCCCAGGGTTGGTCAATTTCGTGCC (2, 1)	871–1250 (390 bp)	⊚	11	NT_009237.18 (N)	10471156–10471538 (383 bp)
	R	GAGCAAGAGGTGGTGAGGTTG (4, 2)		⊚	11	AC021914.7 (L)	43491–43873 (383 bp)
**#4**	F	CCTGGCGGTGCTTCATATCC (6, 2)	1172–1612 (441 bp)	⊚	11	NT_009237.18 (N)	10470794–10471234 (441 bp)
	R	GCTACACTCTGGTTCGTCCAAG (5, 3)		⊚	11	AC021914.7 (L)	43795–44235 (441 bp)
				⊚	5	NT_006713.15 (N)	30541284–30541724 (441 bp)
				⊚	5	AC022223.18 (L)	79289–79727 (441 bp)
**#5**	F	GCCCGTCACCCTCCTCAAG (5, 3)	1485–1950 (466 bp)	⊚	11	NT_009237.18 (N)	10470456–10470921 (466 bp)
	R	ACGGGTGTGCTCTTTTAGCTG (4, 3)		⊚	11	AC021914.7 (L)	44108–44573 (466 bp)
				⊚	3	NT_005612.16 (N)	2831946–2832411 (466 bp)
				⊚	X	AC024033.4 (L)	109808–110273 (466 bp)
**#6**	F	GGAGAGCCAAAGCTAAGACCC (0, 0)	1883–2433 (551 bp)	—	—	—	—
	R	GTGTTGGGTTGACAGTGAGGG (0, 0)					
**#7**	F	GCAGCCACCAATTAAGAAAGCG (15, 7)	2182–2722/541 bp	—	—	—	—
	R	TCTCGTCTTGCTGTGTCATGC (0, 0)					
**#8**	F	AAATTGACCTGCCCGTGAAGAG (4, 5)	2676–3225/550 bp	⊚	17	NT_024862.14 (N)	356823–357372 (550 bp)
	R	CCTGTTCTTGGGTGGGTGTG (1, 3)		⊚	17	NT_024862.13 (L)	340349–340898 (550 bp)
				⊚	17	AF227907.1 (L)	5396–5945 (550 bp)
				⊚	17	AC107940.13 (L)	97187–97736 (550 bp)
**#9**	F	GGAGTAATCCAGGTCGGTT (6, 4)	3079–3505 (427 bp)	—	—	—	—
	R	TAGATGTGGCGGGTTTTAGG (0, 0)					
**#10**	F	GCTACTACAACCCTTCGCTGAC (0, 0)	3438–3893 (456 bp)	—	—	—	—
	R	GTTCGGTTGGTCTCTGCTAGTG (0, 0)					
**#11**	F	CTGCGAGCAGTAGCCCAAAC (0, 0)	3703–4203 (501 bp)	—	—	—	—
	R	TGCTAGGGTGAGTGGTAGGAAG (2, 2)					
**#12**	F	TTCCTACCACTCACCCTAGCA (2, 2)	4183–4552 (370 bp)	⊚	1	NT_004350.19 (N)	43365–43734 (370 bp)
	R	AAAAATCAGTGCGAGCTTAGC (2, 2)		⊚	1	AF134583.1 (L)	270–639 (370 bp)
				⊚	6	AL359496.30 (L)	87935–88304 (370 bp)
**#13**	F	CATCTTTGCAGGCACACTCATC (2, 2)	4505–5003 (499 bp)	△	1	NT_004350.19 (N)	43687–44185 (499 bp)
	R	GATTTTGCGTAGCTGGGTTTGG (0, 0)		△	1	AF134583.1 (L)	592–1090 (499 bp)
				△	6	AL359496.30 (L)	88257–88755 (499 bp)
**#14**	F	CATAGCAGGCAGTTGAGGTGG (0, 0)	4955–5483 (529 bp)	—	—	—	—
	R	AGGTAGGAGTAGCGTGGTAAGG (0, 0)					
**#15**	F	CAAAACCCACCCCATTCCTCC (2, 2)	5428–5926 (499 bp)	⊚	1	NT_004350.19 (N)	44610–45108 (499 bp)
	R	AATAGTCAACGGTCGGCGAAC (2, 2)		⊚	1	AF134583.1 (L)	1515–2013 (499 bp)
				△	6	AL359496.30 (L)	89181–89685 (499 bp)
**#16**	F	ACAGTCCAATGCTTCACTCAGC (4, 3)	5861–6345 (485 bp)	⊚	1	NT_004350.19 (N)	45043–45527 (485 bp)
	R	AGATGGTTAGGTCTACGGAGGC (2, 3)		⊚	1	AF134583.1 (L)	1948–2432 (485 bp)
				⊚	1	AF035429.1 (L)	58–542 (485 bp)
**#17**	F	AGCAGGAACAGGTTGAACAGTC (0, 1)	6266–6669 (404 bp)	△	1	NT_004350.19 (N)	45448–45851 (404 bp)
	R	GGGAGATTATTCCGAAGCCTGG (5, 5)		△	1	AF035429.1 (L)	463–866 (404 bp)
				△	6	AL359496.30 (L)	90025–90428 (404 bp)
**#18**	F	CGGAGGAGGAGACCCCATTC (2, 3)	6572–6831 (260 bp)	⊚	1	NT_004350.19 (N)	45754–46014 (260 bp)
	R	TGGTAGCGGAGGTGAAATATGC (2, 2)		⊚	1	AF134583.1 (L)	2659–2918 (260 bp)
				⊚	1	AF035429.1 (L)	769–1028 (260 bp)
				△	6	AL359496.30 (L)	90331–90590 (260 bp)
**#19**	F	TTCCTAGGGTTTATCGTGTGAGC (2, 3)	6747–7088 (342 bp)	⊚	1	NT_004350.19 (N)	45930–46271 (342 bp)
	R	GTGAATGAAGCCTCCTATGATGG (2, 3)		⊚	1	AF134583.1 (L)	2834–3175 (342 bp)
				⊚	1	AF035429.1 (L)	944–1285 (342 bp)
				⊚	6	AL359496.30 (L)	90506–90847 (342 bp)
**#20**	F	GGTGGCCTGACTGGCATTG (2, 3)	6954–7491 (538 bp)	⊚	1	NT_004350.19 (N)	46137–46674 (538 bp)
	R	GTTGGCTTGAAACCAGCTTTGG (2, 3)		⊚	1	AF134583.1 (L)	3041–3578 (538 bp)
				⊚	1	AF035429.1 (L)	1151–1688 (538 bp)
				⊚	6	AL359496.30 (L)	90713–91250 (538 bp)
**#21**	F	ACCCTACCACACATTCG (3, 4)	7403–7682 (280 bp)	⊚	1	NT_004350.19 (N)	46586–46865 (280 bp)
	R	GGAAAATGATTATGAGGGCG (2, 3)		⊚	1	AF134583.1 (L)	3490–3769 (280 bp)
				⊚	1	AF035429.1 (L)	1600–1879 (280 bp)
				⊚	6	AL359496.30 (L)	9116291441 (280 bp)
**#22**	F	ACAAGACGCTACTTCCCCTATC (2, 2)	7612–8091 (480 bp)	⊚	1	NT_004350.19 (N)	46795–47274 (480 bp)
	R	CCTAATGTGGGGACAGCTCATG (4, 4)		⊚	1	AF134583.1 (L)	3699–4178 (480 bp)
				□	6	AL359496.30 (L)	91371–91850 (480 bp)
**#23**	F	AGTACTCCCGATTGAAGCCCC (0, 0)	8011–8560 (550 bp)	—	—	—	—
	R	GGGCAATGAATGAAGCGAACAG (1, 0)					
**#24**	F	ACCTACCTCCCTCACCAAAGC (2, 2)	8466–8925 (460 bp)	□	1	NT_004350.19 (N)	47647–48106 (460 bp)
	R	TGTGCCTTGTGGTAAGAAGTGG (2, 2)		□	1	AF134583.1 (L)	4551–5010 (460 bp)
				□	6	AL359496.30 (L)	92225–92684 (460 bp)
**#25**	F	GCGGGCACAGTGATTATAGG (0, 0)	8854–9076 (223 bp)	▾	1	NT_004350.19 (N)	48035–48257 (223 bp)
	R	TGGTTGATATTGCTAGGGTGGC (0, 0)		#	1	AF134583.1 (L)	4939–5162 (223 bp)
				▾	6	AL359496.30 (L)	9261392835 (223 bp)
**#26**	F	CGCCTAACCGCTAACATTACTG (2, 1)	9001–9335 (335 bp)	—	—	—	—
	R	GAGGAGCGTTATGGAGTGGAAG (0, 0)					
**#27**	F	TCTCAGCCCTCCTAATGACCTC (1, 1)	9271–9793 (523 bp)	—	—	—	—
	R	GTTGAGCCGTAGATGCCGTC (0, 0)					
**#28**	F	CAGAGTACTTCGAGTCTCCCTTC (0, 0)	9742–9988 (247 bp)	—	—	—	—
	R	GACCCTCATCAATAGATGGAGAC (0, 0)					
**#29**	F	ATCAACACCCTCCTAGCCTTAC (0, 0)	10083–10407 (325 bp)	—	—	—	—
	R	CCAATTCGGCTCAGTCTAATCC (2, 0)					
**#30**	F	CCCTACCATGAGCCCTACAAAC (0, 0)	10279–10634 (356 bp)	▽	5	NM_001838952.21 (N)	39403487–39403839 (356 bp)
	R	TAAGAGGGAGTGGGTGTTGAGG (1, 3)		▽	5	AC008670.6 (L)	8174382013 (356 bp)
**#31**	F	TCGCTCACACCTCATATCCTCC (0, 0)	10535–10734 (200 bp)	▴	5	NT_34772.6 (N)	42577624–42577823 (200 bp)
	R	AGTCTAGGCCATATGTGTTGGAG (0, 0)		▴	5	AC008670.6 (L)	81994–82193 (200 bp)
**#32**	F	GCCTAGCCCTACTAGTCTCAATC (2, 3)	10690–10925 (236 bp)	—	—	—	—
	R	AGGTTGGGGAACAGCTAAATAGG (0, 0)					
**#33**	F	ATCAACACAACCACCCACAGC (2, 1)	10832–11315 (484 bp)	▴	5	NT_34772.6 (N)	42577043–42577526 (484 bp
	R	GTTCTTGGGCAGTGAGAGTGAG (0, 0)		▴	5	AC008670.6 (L)	82291–82274 (484 bp)
**#34**	F	TGAACGCAGGCACATACTTCC (0, 0)	11187–11719 (533 bp)	#	5	NT_34772.6 (N)	42576639–42577171 (533 bp)
	R	GCCGTGGGCGATTATGAGAATG (0, 0)		#	5	AC021965.3 (L)	94060–94592 (533 bp)
**#35**	F	ACAGCCATTCTCATCCAAACCC (0, 0)	11654–12195 (542 bp)	▴	5	NW_001838952.2 (N)	4525902–4526443 (542 bp)
	R	GGTCGTAAGCCTCTGTTGTCAG (0, 0)		▴	5	AC008670.6 (L)	83113–83654 (542 bp)
**#36**	F	GCTCACTCACCCACCACAT (0, 0)	12009–12462 (454 bp)	—	—	—	—
	R	GGATGCGACAATGGATTTTA (0, 0)					
**#37**	F	ACCACCCTAACCCTGACTTCC (0, 0)	12358–12848 (491 bp)	—	—	—	—
	R	GCTTGAATGGCTGCTGTGTTG (0, 0)					
**#38**	F	GATGATACGCCCGAGCAGATG (2, 1)	12806–13311 (506 bp)	□	5	NW_001838952.2 (N)	4527054–4527559 (506 bp)
	R	TGCTAGGTGTGGTTGGTTGATG (2, 1)		□	5	AC008670.6 (L)	84265–84770 (506 bp)
**#39**	F	TCCACTTCAAGTCAACTAGGAC (0, 0)	13249–13785 (537 bp)	—	—	—	—
	R	GGGGATTGTTGTTTGGAAGGG (0, 0)					
**#40**	F	GCAGCCGGAAGCCTATTCG (0, 0)	13708–14070 (363 bp)	—	—	—	—
	R	TGAGGTGATGATGGAGGTGGAG (0, 0)					
**#41**	F	ATCACACACCGCACAATCCC (0, 0)	13930–14371 (442 bp)	—	—	—	—
	R	ATTGGTGCTGTGGGTGAAAGAG (0, 0)					
**#42**	F	TCCTCCCGAATCAACCCTGAC (0, 0)	14261–14706 (446 bp)	—	—	—	—
	R	TCATTGGTCGTGGTTGTAGTCC (1, 2)		#	5	NT_34772.6 (N)	7695705–7696149 (445 bp)
**#43**	F	AATAACACACCCGACCACAC (0, 0)	14548–14992 (445 bp)	#	5	AC021965.3 (L)	97423–97867 (445 bp)
	R	AAGGTAGCGGATGATTCAGC (2, 1)					
**#44**	F	TCATCAATCGCCCACATCACTC (0, 0)	14936–15341 (406 bp)	—	—	—	—
	R	ATAGGAGGTGGAGTGCTGCTAG (0, 0)					
**#45**	F	AGACAGTCCCACCCTCACAC (0, 0)	15256–15743 (488 bp)	—	—	—	—
	R	GGAGGTCTGCGGCTAGGAG (0, 0)					
**#46**	F	CTCCGATCCGTCCCTAACAAAC (0, 0)	15587–16185 (599 bp)	—	—	—	—
	R	GGTTTTGATGTGGATTGGGT (0, 0)					
**#47**	F	ACATTACTGCCAGCCACCATG (0, 0)	16098–16456 (359 bp)	—	—	—	—
	R	CCGGAGCGAGGAGAGTAGC (0, 0)					
**#48**	F	CAGTCAAATCCCTTCTCGTCCC (0, 0)	16344–276 (502 bp)	—	—	—	—
	R	TCTGTGTGGAAAGTGGCTGTG (0, 0)					

The amplicon numbers (Amp. #) and primer sequences were obtained from the paper of Meierhofer et al [7]. F and R represent forward primer and reverse primer of each amplicon, respectively. Hits on NCBI and Hits on BioEdit indicate the number of hits with perfect match from the primer sequences with nDNA sequences in the GenBank database of the NCBI and the local database created in BioEdit, respectively, according to the BLAST search results. The possibility of 2 primers for each amplicon matching with nDNA on the same chromosome was evaluated, and the matched accession numbers, positions on the chromosome, and the sizes of the putative NUMTs are indicated. Match for NUMT location refers to the conditions of matching both primers to the same chromosome, with the following symbols indicating the matching conditions of the primers to nDNA: ⊚, perfect match for both primers to the accession number indicated; △, perfect match for one primer and one mismatch for another primer; ▴, one mismatch for both primers; ▽, perfect match for one primer and 2 or more mismatches for another primer; ▾, one mismatch for one primer and 2 or more mismatches for another primer; #, 2 or more mismatches for both primers. N and L indicate results from NCBI and local database searches, respectively. The symbol of — indicates that no matching result for the same chromosome could be found. Although some primers could match the same chromosome, even with 2 or more mismatches, the matched primers could be positioned too far away from each other to practically generate PCR products. In such cases, the matching results are not listed.

### Evaluation of the Potential of the SB Primer Set for Amplifying NUMTs by Bioinformatic Analysis and PCR with the DNA of 143B-ρ°Cells

We further performed the same evaluations on the 17 SB primer pairs, which generated long amplicons of approximately 2 kb. Results showed that 9 primer pairs exhibited high potential for matching with NUMTs, although only 2 primer pairs, SB#5 and SB#13, perfectly matched the same chromosome (Table 2). These primer pairs could potentially match sequences on chromosomes 1, 6, 8, 17, and X, with highest frequency for chromosomes 1, 5, and 6, which was identical to our observation in the DM primers. In contrast to our findings in the DM primer pairs, the lengths of several NUMTs matched by SB primer pairs slightly differed from those of the targeted mtDNA sequences. We further selected the primer pairs for SB#2, SB#5, SB#7, SB#8, SB#10, SB#12, SB#13, and SB#15 to conduct PCR analyses. As shown in Figure 4B, our results confirmed that the selected primer pairs could amplify the NUMTs because all of the selected SB primers could generate PCR products from the DNA of 143B-ρ^0^ cells. The sizes of these PCR products were very close to those from 143B cells, although the amounts of the PCR products were relatively lower for amplicons SB#5, SB#8, and SB#15.

**Table 2:** Results for the bioinformatics analysis on the matching of SB primers to NUMTs.

Amp. #	Primer sequences and the number of perfect matches with nDNA (Hits on NCBI, Hits on BioEdit)		Amplified mtDNA region (size)	NUMT location for the primer pair that matches to the same chromosome			
				Match	Chr. #	Accession #	Position (size)
**#1 (A)**	F	GATCACAGGTCTATCACCCTA (0, 0)	1–2027 (2027 bp)	✗	—	—	—
	R	TTGGACAACCAGCTATCACCA (22, 18)					
**#2 (B)**	F	GCACACCCGTCTATGTAGCA (4, 3)	1941–3948 (2008 bp)	▾	17	NT_024862.14 (N)	356087–358091 (2005 bp)
	R	TTCGATGTTGAAGCCTGAGAC (6, 3)		▾	17	NT_024862.13 (L)	339613–341617 (2005 bp)
				▾	17	AF227907.1 (L)	4660–6664 (2005 bp)
				▾	17	AC107940.13 (L)	96451–98455 (2005 bp)
**#3 (C)**	F	CCACACTAGCAGAGACCAAC (0, 0)	3869–5883 (2015 bp)	—	—	—	—
	R	GGCTGAGTGAAGCATTGGACT (4, 3)					
**#4 (D)**	F	GAAGCTGCTTCTTCGAATTTGC (5, 2)	5777–7667 (1891 bp)	—	—	—	—
	R	GGGCGTGATCATGAAAGGTG (0, 0)					
**#5 (E)**	F	CAAGTAGGTCTACAAGACGCT (4, 3)	7601–9627 (2027 bp)	⊚	1	NT_004350.19 (N)	46784–48808 (2025 bp)
	R	CTGATGCGAGTAATACGGATG (1, 2)		⊚	1	AF134583.1 (L)	3688–5712 (2025 bp)
				△	5	NT_034772.6 (N)	7701074–7703100 (2027 bp)
				△	5	AC021965.3 (L)	91360–93386 (2027 bp)
				⊚	6	AL359496.30 (L)	90471–92497 (2027 bp)
**#6 (F)**	F	TACCACTCCAGCCTAGCCC (2, 4)	9510–11593 (2084 bp)	△	5	AC021965.3 (L)	92381–94466 (2086 bp)
	R	TCGTAGGCAGATGGAGCTTG (2, 1)		▽		NT_034772.6 (N)	
**#7 (G)**	F	CGGCTATGGTATAATACGCCT (2, 1)	11476–13581 (2106 bp)	▽	5	AC008670.6 (L)	82935–85039 (2106 bp)
	R	AGCGATGAGAGTAATAGATAGG (2, 0)		▽	5	NT_034772.6 (N)	42572871–42574867 (1997 bp)
**#8 (H)**	F	CCTCACAGGTTTCTACTCCAA (1, 1)	13491–15493 (2003 bp)	▽	5	AC008670.6 (L)	84950–86946 (1997 bp)
	R	GAGGTCTGGTGAGAATAGTGT (0, 0)					
**#9 (H')**	F	GCAGCCCTAGCAACACTCC (0, 0)	15314–90 (1346 bp)	—	—	—	—
	R	CAATGCTATCGCGTGCATACC (0, 0)					
**#10 (I)**	F	GAACACACAATAGCTAAGACCC (8, 2)	1045–3079 (2035 bp)	▾	8	NT_167187.1 (N)	20727551–20729527 (1977 bp)
	R	CGGTCTGAACTCAGATCACGTA (3, 1)		▾	8	NT_007995.8 (L)	190554–192530 (1977 bp)
				▾	X	AL158819.14 (L)	2580–4588 (2007 bp)
**#11 (J)**	F	CGATGTTGGATCAGGACATCC (17, 11)	2988–5061 (2073 bp)	—	—	—	—
	R	GGTTGTACGGTAGAACTGCTA (0, 1)					
**#12 (K)**	F	CATAGCAGGCAGTTGAGGTG (0, 0)	4955–7048 (2094 bp)	▽	1	NT_004350.19 (N)	44137–46231 (2095 bp)
	R	GATAGGACATAGTGGAAGTGG (2, 3)		▽	1	NW_001838563.2 (N)	2027–4120 (2095 bp)
				▽	1	AF134583.1 (L)	1042–3135 (2095 bp)
				▽	6	AL359496.30 (L)	88711–90807 (2097 bp)
**#13 (L)**	F	CTCATCACTAGACATCGTACTA (3, 4)	6983–9027 (2045 bp)	⊚	1	NT_004350.19 (N)	46166–48208 (2043 bp)
	R	GCCTGCAGTAATGTTAGCGG (1, 2)		▽	1	NW_001838563.2 (N)	5–−2092 (2043 bp)
				⊚	1	AF134583.1 (L)	3070–5112 (2043 bp)
				⊚	6	AL359496.30 (L)	90742–92786 (2045 bp)
**#14 (M)**	F	CATCAGCCTACTCATTCAACC (4, 3)	8964–10740 (1777 bp)	—	—	—	—
	R	GTACGTAGTCTAGGCCATATG (1, 2)					
**#15 (N)**	F	GCCTAGCCCTACTAGTCTCAA (2, 3)	10690–12769 (2080 bp)	▾	5	NT_034772.6 (N)	7697929–7700008 (2080 bp)
	R	CTCAGCCGATGAACAGTTGG (0, 0)		▾	5	AC021965.3 (L)	93563–95642 (2080 bp)
**#16 (O)**	F	CGTTACATGGTCCATCATAGAA (0, 0)	12621–14700 (2080 bp)	—	—	—	—
	R	GTCGTGGTTGTAGTCCGTGC (1, 2)					
**#17 (P)**	F	CTCCTCAATAGCCATCGCTG (2, 1)	14462–1045 (3193 bp)	—	—	—	—
	R	GGGTCTTAGCTATTGTGTGTTC (5, 2)					

The 17 SB amplicons were designated as A to P amplicons by Bannwarth et al., but we have reassigned it to SB#1 to SB#17 for better illustration. Amp. # represents amplicon #. Primer sequences were obtained from the paper of Bannwarth et al. [11]. F and R represent forward primer and reverse primer of each amplicon, respectively. Hits on NCBI and Hits on BioEdit indicate the number of hits with perfect match from the primer sequences with nDNA sequences in the GenBank database of the NCBI and the local database created in BioEdit, respectively, according to the BLAST search results. The possibility of 2 primers for each amplicon matching with nDNA on the same chromosome was evaluated, and the matched accession numbers, positions on the chromosome, and the sizes of the putative NUMTs are indicated. Match for NUMT location refers to the conditions of matching both primers to the same chromosome, with the following symbols indicating the matching conditions of the primers to nDNA: ⊚, perfect match for both primers to the accession number indicated; △, perfect match for one primer and one mismatch for another primer; ▽, perfect match for one primer and 2 or more mismatches for another primer; ▾, one mismatch for one primer and 2 or more mismatches for another primer. N and L indicate results from NCBI and local database searches, respectively. The symbol of — indicates that no matching result for the same chromosome could be found. Although some primers could match the same chromosome, even with 2 or more mismatches, the matched primers could be positioned too far away from each other to practically generate PCR products. In such cases, the matching results are not listed.

### Demonstration on the Purity of mtDNA Isolated from the Mitochondria of Cultured Cells

**Figure 5:**
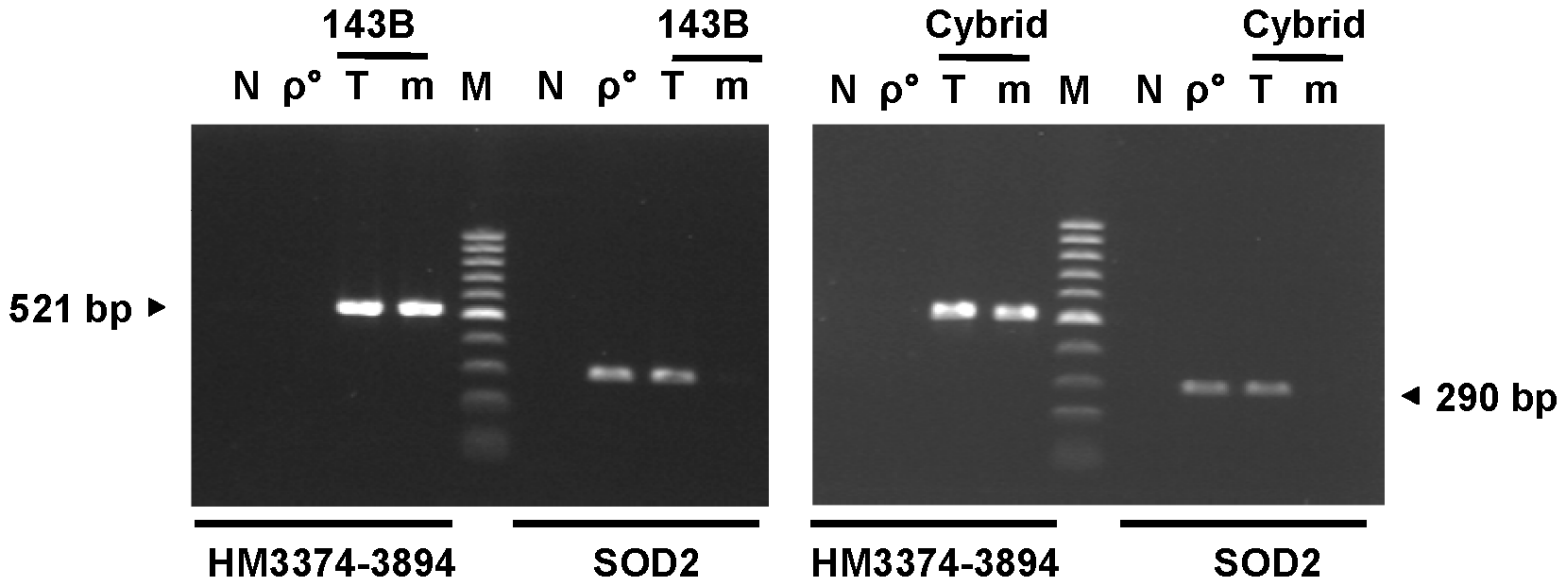
Confirmation of the purity of isolated mtDNA from 143B cells and the cybrid cells. The purity of isolated mtDNA was determined by comparing the PCR results obtained using mtDNA-specific and nDNA-specific primers for DNA from isolated mitochondria, 143B-ρ^0^ cells, 143B cells, and the cybrid cells. The primer pair for the amplicon 337439-bp and 190-bp 3894 of human mtDNA (HM3374–3894) was used as the mtDNA-specific primers, which generated PCR products with an expected size of 521 bp. One primer pair for the *SOD2* gene was used as the nDNA-specific primers, which generated PCR products with an expected size of 290 bp. N, no-template control; ρ^0^, DNA of 143B-ρ^0^ cells as the template; T, total DNA from whole cells as the template; m, mtDNA extracted from isolated mitochondria as the template.

To clearly differentiate true NUMT effects from real mtDNA heteroplasmy or possible PCR artifacts, it was essential to compare the results from total DNA isolated from whole cells with those from pure mtDNA isolated from mitochondrial fraction for suspected primer pairs. To achieve this objective, it was necessary to obtain mtDNA without nDNA contamination, and to verify the purity of the isolated mtDNA. As shown in Figure 5, the primer pair for the 3374–3389 amplicon of mtDNA was mtDNA-specific because it could amplify targeted mtDNA sequences from the total DNA of 143B cells or cybrid cells, or the mtDNA of isolated mitochondria, but not the DNA of 143B-ρ^0^ cells. When we used the primer pair for the nDNA-encoded *SOD2* gene, the DNA of 143B-ρ^0^ cells, 143B cells, or cybrid cells could be amplified, whereas the DNA from isolated mitochondria could not. These examinations proved that the mtDNA purified using our procedures did not have nDNA contamination.

### Assessment of the Effects of NUMTs from the DM Primer Set on mtDNA Detection during DHPLC and SN/WAVE-HS Analysis

We evaluated the DM primer pairs displayed in Figure 4A, except DM#8, by comparing the results for total DNA and those for isolated mtDNA of the same cells analyzed in the same rum. Each DNA sample was subjected to heat-annealing reactions. Among the 16 amplicons tested, 8 amplicons displayed small, but clear, heteroduplexes on the chromatograms of DHPLC analysis at certain temperatures for 143B cells, whereas 4 amplicons displayed ambiguous shoulder peaks adjacent to the major homoduplexes. However, we observed identical patterns for the mtDNA of 143B cells (chromatographic data not shown). Similar results could be found when using 143B cybrid cells, although the optimal analyzing temperatures and the amplicons with clear heteroduplex peaks differed for a few amplicons because of different mtDNA background (chromatographic data not shown). However, sequencing results did not reveal any obvious heteroplasmic sites for these amplicons with heteroduplex signal detected during DHPLC analysis (data not shown). Therefore, we could not confirm the interference of NUMTs on the interpretation of the results of DHPLC analysis for these amplicons and primer pairs. Table 3 lists the results from these evaluations.

**Table 3:** Summary on the evaluation of DM primers during DHPLC and SN/WAVE-HS analyses.

	DHPLC		SN/WAVE-HS			
	143B	Cybrid	143B		Cybrid	
**DM amplicon**			**Uncut**	**Cut vs. Uncut**	**Uncut**	**Cut vs. Uncut**
**#3**	**Y**	**Y**	N	N	ND	ND
**#4**	**Y**	**Y**	**Y**	**Y**	**Y**	**Y**
**#5**	**Y**	**Y**	N	N	ND	ND
**#12**	N	ND	N	N	ND	ND
**#13**	?	**Y**	N	N	ND	ND
**#15**	**Y**	**Y**	**Y**	**Y**	**Y**	**Y**
**#16**	**Y**	?	**Y**	**Y**	**Y**	**Y**
**#17**	**Y**	?	N	N	ND	ND
**#18**	N	ND	N	N	ND	ND
**#19**	?	?	N	N	ND	ND
**#20**	**Y**	**Y**	N	N	ND	ND
**#21**	N	ND	N	N	ND	ND
**#22**	**Y**	**Y**	**Y**	**Y**	**Y**	**Y**
**#24**	?	**Y**	N	N	ND	ND
**#30**	N	ND	N	N	ND	ND
**#38**	?	?	**Y**	**Y**	**Y**	**Y**

For DHPLC analysis, Y indicates the presence of small but clear heteroduplex peaks, whereas N indicates the absence of such peaks. The question mark indicates the presence of ambiguous shoulder peaks. For SN/WAVE-HS analysis, Y in the uncut groups indicates the presence of cleaved DNA fragments of sizes different from those of the undigested peak, whereas Y in the cut vs. uncut column indicates changes in patterns of peaks from cleaved fragments after SN digestion. ND indicates that the experiments were not performed for cybrid cells because there was no positive signal or change in the chromatograms of 143B cells.

**Figure 6:**
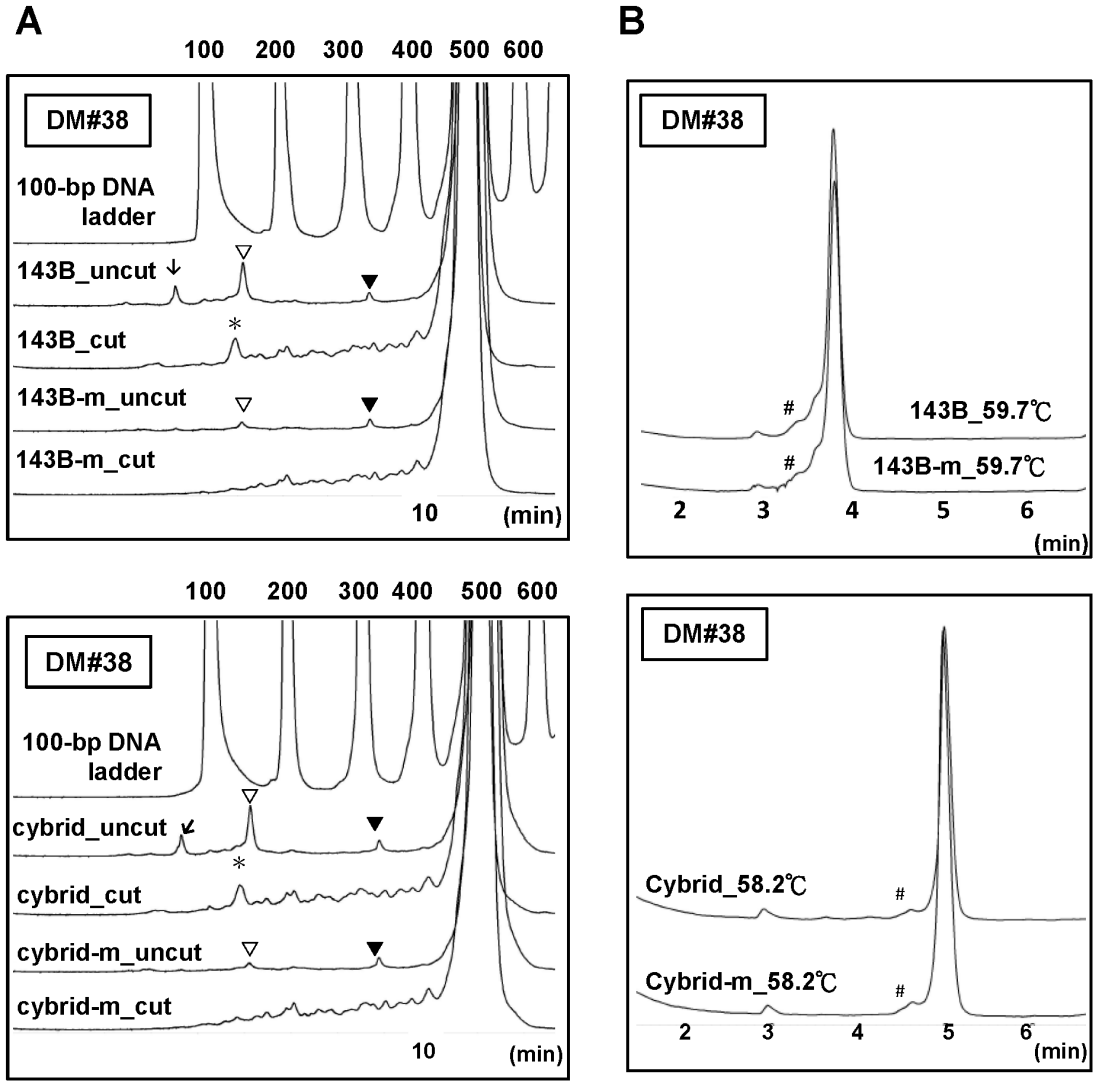
Results from SN/WAVE-HS and DHPLC analysis of the DM#38 amplicon by using total DNA and mtDNA from cells. (A) Results from SN/WAVE-HS analysis. (B) Results from DHPLC analysis. The upper and lower panels of Figure 6A and Figure 6B display the results for 143B cells and the cybrid cells, respectively. In Figure 6A, the results for the DNA samples without (uncut) and with (cut) SN digestion were also compared. ▾ indicates the peaks that appeared in the chromatograms of uncut DNA but were not observed in the chromatograms of the corresponding cut DNA for all DNA samples. ▽ indicates the peaks >100 bp only present in the chromatograms of the uncut DNA of both total DNA and mtDNA (m), but not the cut DNA. Such peaks for the mtDNA were considerably smaller than those of total DNA. ↓ indicates the peaks <100 bp that were present in the chromatograms of the uncut total DNA but absent in those of the uncut mtDNA. The patterns of peaks in the chromatograms from uncut DNA and cut DNA displayed marked differences. The chromatograms of the cut DNA samples displayed numerous small peaks. One obvious peak (indicated by *) appeared for the cut DNA of whole cells but was absent for mtDNA. In Figure 6B, the small but identifiable heteroduplex peaks detected by DHPLC are indicated by #.

The same evaluation was then applied for SN/WAVE-HS analysis. It is important to emphasize that the SN/WAVE-HS analysis not only can detect heteroduplexes and predict approximate cleavage site by comparing the patterns prior to (uncut) and after (cut) SN digestion, but also can detect PCR products of different sizes. Our results for the total DNA of 143B cells showed that among the 16 DM amplicons analyzed, 5 amplicons displayed clear peaks other than the major uncut peaks in uncut groups and also obvious heteroduplexes, indicated by the changes in the patterns of peaks before and after SN digestion. However, except for DM#38, the same peaks or patterns of changes were also found when analyzing the mtDNA of 143B cells (chromatographic data not shown). We also analyzed these 5 amplicons from the cybrid cells. Except for DM#38, the chromatograms for the total DNA and the mtDNA of cybrids cells were also identical for each amplicon, but the patterns of several amplicons were different from those of 143B cells (chromatographic data not shown). Table 3 lists the summary of these findings and Figure 6A displays the chromatograms for DM#38, which was a reprehensive result from 2 repeated experiments. We observed one peak <100 bp in the uncut groups in the total DNA, but not the mtDNA, for amplicon DM#38 of the 143B and cybrid cells. The peak height of another large peak of 100–200 bp for the uncut groups of the total DNA was decreased to near the background level of the uncut groups of the mtDNA from both types of cells. Moreover, after SN digestion, the shift of the 100–200 bp peak to a smaller peak for the total DNA of the 143B and cybrid cells did not occur for the mtDNA. These results demonstrated the interference from NUMTs on SN/WAVE-HS analysis of DM#38 in both the 143B and cybrid cells. In contrast, as shown in Figure 6B, the differences between the total DNA and the mtDNA in DHPLC analysis of 143B cells or the cybrid cells were absent, although the chromatograms for the total DNA and the mtDNA both displayed ambiguous shoulder peaks adjacent to the homoduplex peaks.

### Assessment of the Effects of NUMTs from the SB Primer Set on mtDNA Detection during SN/WAVE-HS Analysis

**Figure 7:**
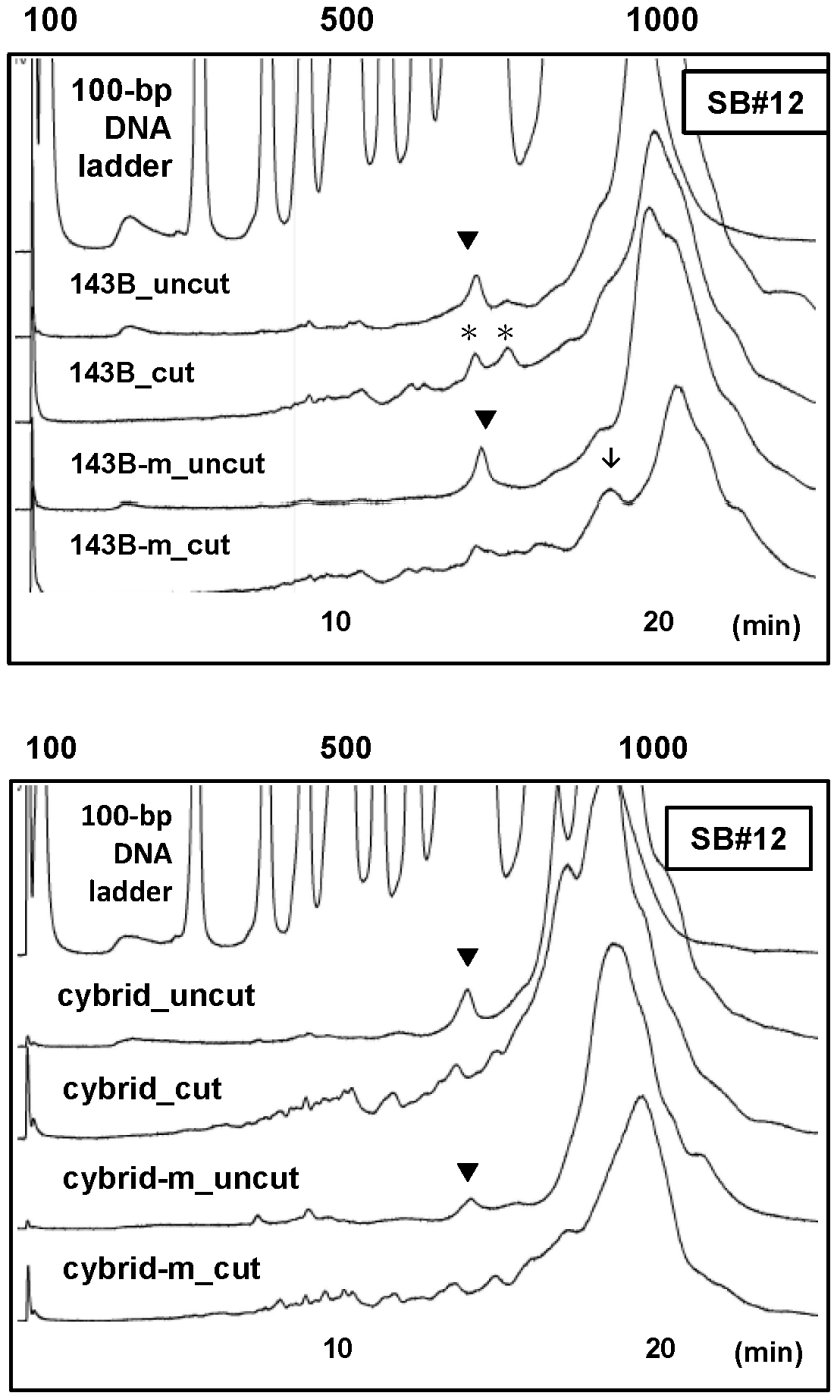
Results from SN/WAVE-HS analysis of the SB#12 amplicon by using total DNA and mtDNA from cells. DNA samples from isolated mitochondria (m) and whole cells of 143B cells and the cybrid were used. The upper and lower panels display the results for 143B cells and the cybrid cells, respectively. The results for the DNA samples without (uncut) and with (cut) SN digestion were also compared. ▾ indicates the peaks that appeared in the chromatograms of uncut DNA but were absent in the chromatograms of the corresponding cut DNA for all DNA samples. Two obvious peaks (indicated by *) showed up after SN digestion for the total DNA of 143B cells, but could not be apparently visualized for the mtDNA of 143B cells (upper panel).↓ indicates the peak close to 1000 bp in the cut DNA of the mtDNA, but not in the cut DNA of the total DNA from 143B cells (upper panel). These two differences in the patterns of peaks between total DNA and mtDNA found in 143B cells were absent in the cybrid cells (lower panel).

The same strategy was further applied to evaluate selected SB primer pairs during SN/WAVE-HS analysis. It was not tested for DHPLC analysis because SB amplicons were too long to be analyzed by DHPLC. After SN digestion, all tested amplicons produced several peaks in low abundance in the total DNA of the 143B or cybrid cells, but the patterns were highly similar to those of the mtDNA (data not shown) except SB#12. A representative data from 2 repeated experiments were displayed in Figure 7. As shown in the upper panel of Figure 7, there was a dominant peak of approximately 800 bp in the uncut groups for the total DNA and the mtDNA from 143B cells, but 2 peaks at the similar retention time showed up in the cut group of the total DNA, but not the mtDNA. On the contrary, we observed a peak close to 1000 bp in the chromatogram for the mtDNA, but not the total DNA, of 143B cells. These findings indicated the contributions of NUMTs to such differences. However, the results displayed in the lower panel of Figure 7 indicated that the patterns of peaks in the cut groups did not exhibit obvious differences between the total DNA and the mtDNA of the cybrid cells.

### Sequencing Results for the DM#38 and SB#12 Amplicons of 143B-ρ°Cells, 143B Cells, and the Cybrid Cells

To examine whether sequencing data of different cells could be used to explain the findings for the DM#38 and SB#12 amplicons during SN/WAVE-HS analysis, PCR products for these 2 amplicons from 143B-ρ^0^ cells, 143B cells, and the cybrid cells were sequenced. The differences in the sequences among these 3 cell lines for the nt 12806–13311 (DM#38) and nt 4995–7048 (SB#12) regions were listed in Table S1 and Table S2, respectively. Our sequencing results showed that the mtDNA of the 143B cells and cybrid cells differed only at position 13135 for DM#38, and that mtDNA of these two cell lines had 14 nucleotides with the sequences different from those of 143B-ρ^0^ cells (Table S1). However, although the sequences of the DNA of 143B-ρ^0^ cells also differed from mtDNA sequences of either of the 143B or cybrids at 26 nucleotide positions for SB#12, 6 of 7 nucleotides positions with different mtDNA sequences between 143B cells and the cybrid cells had the same sequences between the mtDNA of the cybrid cells and the DNA of 143B-ρ^0^ cells, but not between the mtDNA of 143B cells and the DNA of 143B-ρ^0^ cells for this long amplicon (Table S2). Our sequencing results might explain the identical NUMT effects observed in 143B cells and the cybrids cells for DM#38, because of one nucleotide difference in the mtDNA background between these 2 cell lines for this region. The clear NUMT effect in 143B cells, but not the cybrid cells, for SB#12 might be caused by closer sequence similarity between nDNA of 143B-ρ^0^ cells and mtDNA of the cybrid cells than between 143B-ρ^0^ cells and 143B cells. However, the chromatographic data of sequencing indicated that the mtDNA sequences in these 2 regions for 143B cells and the cybrid cells displayed no sign of heteroplasmy (data not shown). It was therefore impossible to identify the presence of interference from NUMTs based on the sequencing results for the DM#38 and SB#12 amplicons.

## Discussion

Our current study is the first to apply the SN/WAVE-HS platform for the detection of mtDNA and to compare its performance with that of DHPLC. Our results suggest that the relative sensitivity of the SN/WAVE-HS platform for the detection of mtDNA heteroplasmy is marginally higher than that of DHPLC for one SNP, although both techniques determine heteroplasmy qualitatively. Furthermore, we have provided a practical approach for the screening and evaluation of the potential interference of NUMTs on mtDNA detection during DHPLC and SN/WAVE-HS analysis from primers by using published primer sets as an example. Most importantly, this study is the first to verify NUMTs effects on the determination of mtDNA heteroplasmy by obtaining pure mtDNA from isolated mitochondria without PCR-detectable nDNA contamination.

Both DHPLC and SN/WAVE-HS platforms are qualitative techniques and thus was not suitable to be evaluated for the exact limit of detection. By conducting analysis on a serial of samples with different percentages of mutant, Janne et al. reported that the sensitivity of SN/WAVE-HS method for the EFRF gene reached 1% of mutant [10], which was similar to 2% observed in this study for plasmids with an SNP. We identified that DHPLC was suitable to detect mutant at 5% or greater in this study, which was consistent with that reported in our previous study [4] and in other studies [8], [27] for mtDNA. Because testing on the optimal temperatures required for DHPLC analysis is not conducted for SN/WAVE-HS analysis, and the WAVE HS System is superior to gel electrophoresis for the identification of cleaved DNA fragments with their approximate sizes, theoretically, the SN/WAVE-HS platform should provide a considerably more efficient and convenient method than DHPLC for the screening of mtDNA mutations and heteroplasmy. However, we only tested the template with one mismatch in the amplicon. When there are more than one mismatch in one amplicon, the signals indicating the presence of heteroduplexes and sensitivity should be enhanced during DHPLC analysis, but might be reduced in SN/WAVE-HS analysis due to the formation of many small cleaved fragments, especially that the size of resulting cleaved DNA fragments were recommended to be > 100 bp for the detection in the WAVE HS System. On the other hand, our results suggest that several factors, including non-specific activities of SN, the presence of co-amplified NUMTs, the presence of non-specific PCR products of different sizes prior to SN digestion, potential PCR artifacts, and true low-abundance of mtDNA heteroplasmy that commonly occurs in samples, may cause highly ambiguous signals during SN/WAVE-HS analysis. Such problems may be amplified when working on large amplicons. Therefore, although we did not test amplicons with multiple SNPs from mtDNA of human blood for the SN/WAVE-HS analysis in this study, we suspected that the usefulness of the SN/WAVE-HS platform in the screening or identification of mutations for mtDNA may not be as good as for nDNA.

Although our methods consisting of bioinformatic analysis and PCR verification using the DNA from 143B-ρ^0^ cells indicated the amplification of NUMTs by several primer pairs, only 2 of 24 primer pairs tested experienced interference by NUMTs during SN/WAVE-HS analysis. None of these primers were associated with NUMT effects during DHPLC analysis after comparing the results obtained using pure mtDNA and total DNA for either 143B cells or the cybrid cells. Therefore, low-level heteroduplexes, which could be regarded as low-level heteroplasmy of mtDNA, detected in some amplicons from 143B cells could be true heteroplasmy at any positions in these amplicons. They could be also heteroduplexes and chimerical molecules generated during PCR [28], [29]. One critical implication of our results was that the use of mtDNA deriving from isolated mitochondria without nDNA contamination was essential to prove the interference from NUMTs for a primer pair. However, so far only the study of Calvignac et al. has investigated the feasibility of extracting mtDNA from mitochondrial fraction from the superfamily Aselloidea on preventing contamination of co-amplified NUMTs during PCR, but they simply conducted one low-speed centrifugation followed by one high-speed centrifugation before the DNA extraction of the final pellet without showing any data to demonstrate the purity of isolated mtDNA [22]. In this study, we found that one low-speed centrifugation was not sufficient to fully centrifuge down all nuclear fractions and cell debris, and treatment of the final mitochondrial pellet by DNase I was also critical to eliminate any nDNA attached to the surface of unbroken mitochondria. Moreover, if proper primers and control DNA samples were not used to confirm the purity of isolated mtDNA as we have performed, the conclusion from the results might be misleading. However, our results did not suggest that pure mtDNA from real samples should be obtained during routine analysis of mtDNA mutations when using high-sensitivity techniques because it is not practical for most studies and is generally not applicable in clinical studies. Instead, our study developed a strategy for the evaluation of NUMT effects during primer design by obtaining an “mtDNA control” from unlimited cultured cell lines for confirming NUMT effects before using these primers to analyze real specimen. Our strategy is useful because ρ^0^ cells are not available to most investigators, and primer pairs that can amplify the DNA from ρ^0^ cells, which is often hard to be avoided for some mtDNA regions, such as COI and COII genes with long sequences of high similarity to those of NUMTs, are potentially still usable through the evaluation by using our strategy.

Investigators have created databases for NUMTs by matching entire mtDNA sequences with different human genome databases by applying different criteria [13], [17], and by sequencing cloned PCR product from ρ^0^ cells [30] or isolated nDNA from sperm cells [31] with the use of2 several primer pairs covering all regions of mtDNA. Although those databases may be useful for the screening of primers matchable to NUMTs, the real probability of co-amplification of NUMTs with mtDNA during the PCR by a primer pair and the subsequent effects of NUMTs on the detection of mtDNA variants might not be predictable by using these databases. The reasons are that primer pairs other than the perfectly matched primer pairs are still able to amplify NUMTs, and that the abundances of co-amplified NUMTs are dependent on the copy numbers of NUMTs amplified by each primer pair. For example, in our study, although two primers for the amplicon DM#38, which was associated with the NUMT effects, had perfect matches with the same chromosomes, only the reverse primer for the amplicon SB#12, also associated with the NUMT effects exhibited perfect matches with sequences of multiple accession numbers. However, 16 of 20 bp of the forward primer for SB#12 in fact matched with at least 3 sites in 2 chromosomes without any gap as the reverse primer (data not shown). Another problem is that the NUMT databases produced by the sequencing of cloned PCR products amplified from the DNA of ρ^0^ cells or isolated nDNA by using a set of specific primer pairs might not predict the probability of the co-amplification of NUMTs by a different primer pair. Moreover, the absence of mtDNA in isolated nDNA for such purposes were not demonstrated, such as in the study of Ramos et al., although the authors claimed the purity of the nDNA used [31]. Therefore, the “*in silico* primer validation” approach employed by Ramos et al. [17], which used a database generated from bioinformatics analysis, to make conclusion on the risk of the co-amplification of NUMTs with mtDNA, and to determine whether the reported mtDNA mutations were authentic for the primer pairs used in cancer studies by other studies might mistakenly exclude many true mtDNA mutations reported previously.

When heteroplasmic variants of mtDNA were found to match with nucleotide sequences in NUMT databases, many investigators often inferred that such findings should be caused by co-amplification of NUMTs. However, without appropriate control experiments, particularly when conducting low-sensitivity sequencing, the conclusion could be considered arbitrary. For example, in the study of Parr et al. [30], when PCR products on the agarose gel could be generated from the DNA of both ρ^0^ cells and clinical samples with the same primer pairs, a proportion of the heteroplasmic signals from sequencing were determined to be false results because some identical nucleotide sequences existed in the additional variant and the NUMTs. However, no control DNA, such as the pure mtDNA employed in this study, was tested to verify such conclusion. If nucleotide signals from NUMTs could be so easily detected by the low-sensitivity sequencing technique, as long as the same primer pairs could amplify the DNA of ρ^0^ cells, the interference of NUMTs to the detection of mtDNA heteroplasmy during DHPLC or SN/WAVE-HS analyses would have been tremendous. However, our results showed the probability of such interference was low, despite several primers pairs perfectly matching the nDNA sequences on the same chromosomes.

NUMTs may affect the detection of mtDNA during DHPLC and SN/WAVE-HS analyses in manners additional to the factor of NUMT copy number. Heteroduplex formation between NUMTs and mtDNA, or between different NUMTs, can potentially cause low-level heteroduplex signals during DHPLC analysis and the generation of cleaved DNA fragments after SN digestion. In contrast, signals of heteroduplexes from true mtDNA heteroplasmy might be suppressed because of a dilution effect caused by NUMTs containing the same nucleotide sequences as those of the dominant alleles. Theoretically, the greater the extent of mismatch in one amplicon, the more effective the resolution of the heteroduplex signals during DHPLC analysis, whereas it may make cleaved DNA fragments too small to be detectable during SN/WAVE-HS analysis. Therefore, although we found that low-level heteroduplexes detected by DHPLC for some primer pairs tested in this study were not related to NUMTs, the likehood of generating heteroduplex signals caused by NUMTs during DHPLC analysis when using other untested primer pairs remains high and requires further evaluation. On the other hand, a unique interference from NUMTs that can occur during SN/WAVE-HS analysis, but not during DHPLC analysis, is the detection of putative NUMT products with sizes differing from those of the expected mtDNA amplicons in samples prior to SN digestion. However, non-specific PCR products may also cause these results because the WAVE HS System can detect DNA fragments in different sizes with much greater sensitivity and clarity than agarose gel electrophoresis can. Accordingly, the use of isolated mtDNA is important to clarify this issue. As shown by the results of Figure 6 and Figure 7, peaks present in the chromatograms of both total DNA and mtDNA should be caused by non-specific PCR products. When the patterns of certain peaks were different between the chromatograms of total DNA and mtDNA, NUMTs are likely to play the major roles in such differences. Therefore, the absence of peaks in the uncut and cut samples of mtDNA in comparison with the pattern of total DNA, for DM#38 indicates enhancement of non-specific signals caused by NUMTs (Figure 6). On the other hand, our results suggest that samples with different mtDNA backgrounds are subject to different types of NUMT effects. Consequently, we observed potential interference from NUMTs in both 143B and cybrid cells for the DM#38 amplicon (Figure 6), but only in 143B cells for the SB#12 amplicon (Figure 7). It was possibly because of more differences in the nucleotide sequences between mtDNA of 143B and mtDNA of the cybrid cells, or higher sequence similarity between nDNA of 143B-ρ^0^ cells and mtDNA of the cybrid cells than between 143B-ρ^0^ cells and mtDNA of 143B cells, for the SB#12 amplicon.

Overall, our studies have produced several crucial novel findings. First, the sensitivity of SN/WAVE-HS analysis for the detection of mtDNA heteroplasmy is marginally greater than that of DHPLC analysis for amplicons with one SNP, but several potential factors may affect decision-making on the heteroplasmy or homoplasmy status of mtDNA during SN/WAVE-HS analysis. Second, we results suggest that the use of a bioinformatics approach or the use of the DNA from ρ^0^ cells for the evaluation on the potential amplification of NUMTs by a primer pair is helpful during primer design to exclude some primers with high risk to amplify NUMTs, but is not capable of fully predicting potential interference by NUMTs on the judgment of mtDNA heteroplasmy during the detection by a high-sensitivity and PCR-based technique. Third, mtDNA isolated from the mitochondria of cell lines with confirmed absence of nDNA contamination by PCR is required during primer design to verify NUMT effects from a highly suspected primer pair for subsequent analysis of mtDNA in the total DNA from real samples. Finally, when NUMTs interfere with the judgment of mtDNA heteroplasmy, the extent of such effects may be dependent on the mtDNA backgrounds of samples and the detection methods used.

## Supporting Information

Figure S1 **Simultaneous detection of the same DNA samples used in** **Figure 2B** **by using the UV detector during SN/WAVE-HS analysis.** During the detection of the DNA by the HS detector on the WAVE System, the data acquired by the UV detector of the WAVE System were recorded simultaneously. ▾ indicates the expected DNA fragments in the sizes of 139 bp and 190 bp after SN digestion of heat-annealed PCR products.
(TIFF)

Figure S2 **SN/WAVE-HS analysis of the same PCR products used in** **Figure 2** **by using the old SN kit.** The same heat-annealed PCR products were analyzed by SN/WAVE-HS by using the SURVEYOR Mutation Detection Kit.
(TIF)

Table S1 **Positions in the DM#38 amplicon (nt 12806–13311) with different nucleotide sequences among 143B-ρ^0^ cells, 143B cells, and the cybrid cells.** The mtDNA sequences between 143B and the cybrid cells are only different at position 13135, which is indicated by the bold and italic fonts.
(PDF)

Table S2 **Positions in the SB#12 amplicon (nt 4955–7048) with different nucleotide sequences among 143B-ρ^0^ cells, 143B cells, and the cybrid cells.** The mtDNA sequences between 143B and the cybrid cells are different at several positions, which are indicated by the bold and italic fonts.
(PDF)
