# Correction: Interference of Co-amplified Nuclear Mitochondrial DNA Sequences on the Determination of Human mtDNA Heteroplasmy by Using the SURVEYOR Nuclease and the WAVE HS System

**DOI:** 10.1371/journal.pone.0102209

**Published:** 2014-07-08

**Authors:** The PLOS ONE Staff

There are errors in Tables 1 and 2. In Table 1, all instances of the square symbol (□) should appear as a black dot (•). In Table 2, the x listed in the row for Amp. #1(A) should appear as a dash (—), and the Position value for Amp. #13 should read “50-2092 (2043 bp)”. Please view the corrected tables below.

**Table 1:** Results for the bioinformatics analysis on the matching of DM primers to NUMTs.

Amp. #	Primer sequences and the number of perfect matches with nDNA (Hits on NCBI, Hits on BioEdit)		Amplified mtDNA region (size)	NUMT location for the primer pair that matches to the same chromosome			
				Match	Chr. #	Accession #	Position (size)
**#1**	F	AGCACCCTATGTCGCAGTATC (0, 0)	108—638 (531 bp)	—	—	—	—
	R	GGTGATGTGAGCCCGTCTAAAC (2, 1)					
**#2**	F	CCAACCAAACCCCAAAGAC (4, 2)	548—964 (417 bp)	—	—	—	—
	R	GGGAGGGGGTGATCTAAAAC (0, 0)					
**#3**	F	GCCCCGCCCCAGGGTTGGTCAATTTCGTGCC (2, 1)	871—1250 (390 bp)	•	11	NT_009237.18 (N)	10471156—10471538 (383 bp)
	R	GAGCAAGAGGTGGTGAGGTTG (4, 2)		•	11	AC021914.7 (L)	43491—43873 (383 bp)
**#4**	F	CCTGGCGGTGCTTCATATCC (6, 2)	1172—1612 (441 bp)	•	11	NT_009237.18 (N)	10470794—10471234 (441 bp)
	R	GCTACACTCTGGTTCGTCCAAG (5, 3)		•	11	AC021914.7 (L)	43795—44235 (441 bp)
				•	5	NT_006713.15 (N)	30541284—30541724 (441 bp)
				•	5	AC022223.18 (L)	79289—79727 (441 bp)
**#5**	F	GCCCGTCACCCTCCTCAAG (5, 3)	1485—1950 (466 bp)	•	11	NT_009237.18 (N)	10470456—10470921 (466 bp)
	R	ACGGGTGTGCTCTTTTAGCTG (4, 3)		•	11	AC021914.7 (L)	44108—44573 (466 bp)
				•	3	NT_005612.16 (N)	2831946—2832411 (466 bp)
				•	X	AC024033.4 (L)	109808—110273 (466 bp)
**#6**	F	GGAGAGCCAAAGCTAAGACCC (0, 0)	1883—2433 (551bp)	—	—	—	—
	R	GTGTTGGGTTGACAGTGAGGG (0, 0)					
**#7**	F	GCAGCCACCAATTAAGAAAGCG (15, 7)	2182—2722/541 bp	—	—	—	—
	R	TCTCGTCTTGCTGTGTCATGC (0, 0)					
**#8**	F	AAATTGACCTGCCCGTGAAGAG (4, 5)	2676—3225/550 bp	•	17	NT_024862.14 (N)	356823—357372 (550 bp)
	R	CCTGTTCTTGGGTGGGTGTG (1, 3)		•	17	NT_024862.13 (L)	340349—340898 (550 bp)
				•	17	AF227907.1 (L)	5396—5945 (550 bp)
				•	17	AC107940.13 (L)	97187—97736 (550 bp)
**#9**	F	GGAGTAATCCAGGTCGGTT (6, 4)	3079—3505 (427 bp)	—	—	—	—
	R	TAGATGTGGCGGGTTTTAGG (0, 0)					
**#10**	F	GCTACTACAACCCTTCGCTGAC (0, 0)	3438—3893 (456 bp)	—	—	—	—
	R	GTTCGGTTGGTCTCTGCTAGTG (0, 0)					
**#11**	F	CTGCGAGCAGTAGCCCAAAC (0, 0)	3703—4203 (501 bp)	—	—	—	—
	R	TGCTAGGGTGAGTGGTAGGAAG (2, 2)					
**#12**	F	TTCCTACCACTCACCCTAGCA (2, 2)	4183—4552 (370 bp)	•	1	NT_004350.19 (N)	43365—43734 (370 bp)
	R	AAAAATCAGTGCGAGCTTAGC (2, 2)		•	1	AF134583.1 (L)	270—639 (370 bp)
				•	6	AL359496.30 (L)	87935—88304 (370 bp)
**#13**	F	CATCTTTGCAGGCACACTCATC (2, 2)	4505—5003 (499 bp)	△	1	NT_004350.19 (N)	43687—44185 (499 bp)
	R	GATTTTGCGTAGCTGGGTTTGG (0, 0)		△	1	AF134583.1 (L)	592—1090 (499 bp)
				△	6	AL359496.30 (L)	88257—88755 (499 bp)
**#14**	F	CATAGCAGGCAGTTGAGGTGG (0, 0)	4955—5483 (529 bp)	—	—	—	—
	R	AGGTAGGAGTAGCGTGGTAAGG (0, 0)					
**#15**	F	CAAAACCCACCCCATTCCTCC (2, 2)	5428—5926 (499 bp)	•	1	NT_004350.19 (N)	44610—45108 (499 bp)
	R	AATAGTCAACGGTCGGCGAAC (2, 2)		•	1	AF134583.1 (L)	1515—2013 (499 bp)
				△	6	AL359496.30 (L)	89181—89685 (499 bp)
**#16**	F	ACAGTCCAATGCTTCACTCAGC (4, 3)	5861—6345 (485 bp)	•	1	NT_004350.19 (N)	45043—45527 (485 bp)
	R	AGATGGTTAGGTCTACGGAGGC (2, 3)		•	1	AF134583.1 (L)	1948—2432 (485 bp)
				•	1	AF035429.1 (L)	58—542 (485 bp)
**#17**	F	AGCAGGAACAGGTTGAACAGTC (0, 1)	6266—6669 (404 bp)	△	1	NT_004350.19 (N)	45448—45851 (404 bp)
	R	GGGAGATTATTCCGAAGCCTGG (5, 5)		△	1	AF035429.1 (L)	463—866 (404 bp)
				△	6	AL359496.30 (L)	90025—90428 (404 bp)
**#18**	F	CGGAGGAGGAGACCCCATTC (2, 3)	6572—6831 (260 bp)	•	1	NT_004350.19 (N)	45754—46014 (260 bp)
	R	TGGTAGCGGAGGTGAAATATGC (2, 2)		•	1	AF134583.1 (L)	2659—2918 (260 bp)
				•	1	AF035429.1 (L)	769—1028 (260 bp)
				△	6	AL359496.30 (L)	90331—90590 (260 bp)
**#19**	F	TTCCTAGGGTTTATCGTGTGAGC (2, 3)	6747—7088 (342 bp)	•	1	NT_004350.19 (N)	45930—46271 (342 bp)
	R	GTGAATGAAGCCTCCTATGATGG (2, 3)		•	1	AF134583.1 (L)	2834—3175 (342 bp)
				•	1	AF035429.1 (L)	944—1285 (342 bp)
				•	6	AL359496.30 (L)	90506—90847 (342 bp)
**#20**	F	GGTGGCCTGACTGGCATTG (2, 3)	6954—7491 (538 bp)	•	1	NT_004350.19 (N)	46137—46674 (538 bp)
	R	GTTGGCTTGAAACCAGCTTTGG (2, 3)		•	1	AF134583.1 (L)	3041—3578 (538 bp)
				•	1	AF035429.1 (L)	1151—1688 (538 bp)
				•	6	AL359496.30 (L)	90713—91250 (538 bp)
**#21**	F	ACCCTACCACACATTCG (3, 4)	7403—7682 (280 bp)	•	1	NT_004350.19 (N)	46586—46865 (280 bp)
	R	GGAAAATGATTATGAGGGCG (2, 3)		•	1	AF134583.1 (L)	3490—3769 (280 bp)
				•	1	AF035429.1 (L)	1600—1879 (280 bp)
				•	6	AL359496.30 (L)	9116291441 (280 bp)
**#22**	F	ACAAGACGCTACTTCCCCTATC (2, 2)	7612—8091 (480 bp)	•	1	NT_004350.19 (N)	46795—47274 (480 bp)
	R	CCTAATGTGGGGACAGCTCATG (4, 4)		•	1	AF134583.1 (L)	3699—4178 (480 bp)
				•	6	AL359496.30 (L)	91371—91850 (480 bp)
**#23**	F	AGTACTCCCGATTGAAGCCCC (0, 0)	8011—8560 (550 bp)	—	—	—	—
	R	GGGCAATGAATGAAGCGAACAG (1, 0)					
**#24**	F	ACCTACCTCCCTCACCAAAGC (2, 2)	8466—8925 (460 bp)	•	1	NT_004350.19 (N)	47647—48106 (460 bp)
	R	TGTGCCTTGTGGTAAGAAGTGG (2, 2)		•	1	AF134583.1 (L)	4551—5010 (460 bp)
				•	6	AL359496.30 (L)	92225—92684 (460 bp)
**#25**	F	GCGGGCACAGTGATTATAGG (0, 0)	8854—9076 (223 bp)	▾	1	NT_004350.19 (N)	48035—48257 (223 bp)
	R	TGGTTGATATTGCTAGGGTGGC (0, 0)		#	1	AF134583.1 (L)	4939—5162 (223 bp)
				▾	6	AL359496.30 (L)	9261392835 (223 bp)
**#26**	F	CGCCTAACCGCTAACATTACTG (2, 1)	9001—9335 (335 bp)	—	—	—	—
	R	GAGGAGCGTTATGGAGTGGAAG (0, 0)					
**#27**	F	TCTCAGCCCTCCTAATGACCTC (1, 1)	9271—9793 (523 bp)	—	—	—	—
	R	GTTGAGCCGTAGATGCCGTC (0, 0)					
**#28**	F	CAGAGTACTTCGAGTCTCCCTTC (0, 0)	9742—9988 (247 bp)	—	—	—	—
	R	GACCCTCATCAATAGATGGAGAC (0, 0)					
**#29**	F	ATCAACACCCTCCTAGCCTTAC (0, 0)	10083—10407 (325 bp)	—	—	—	—
	R	CCAATTCGGCTCAGTCTAATCC (2, 0)					
**#30**	F	CCCTACCATGAGCCCTACAAAC (0, 0)	10279—10634 (356 bp)	▽	5	NM_001838952.21 (N)	39403487—39403839 (356 bp)
	R	TAAGAGGGAGTGGGTGTTGAGG (1, 3)		▽	5	AC008670.6 (L)	8174382013 (356 bp)
**#31**	F	TCGCTCACACCTCATATCCTCC (0, 0)	10535—10734 (200 bp)	▴	5	NT_34772.6 (N)	42577624—42577823 (200 bp)
	R	AGTCTAGGCCATATGTGTTGGAG (0, 0)		▴	5	AC008670.6 (L)	81994—82193 (200 bp)
**#32**	F	GCCTAGCCCTACTAGTCTCAATC (2, 3)	10690—10925 (236 bp)	—	—	—	—
	R	AGGTTGGGGAACAGCTAAATAGG (0, 0)					
**#33**	F	ATCAACACAACCACCCACAGC (2, 1)	10832—11315 (484 bp)	▴	5	NT_34772.6 (N)	42577043—42577526 (484 bp
	R	GTTCTTGGGCAGTGAGAGTGAG (0, 0)		▴	5	AC008670.6 (L)	82291—82274 (484 bp)
**#34**	F	TGAACGCAGGCACATACTTCC (0, 0)	11187—11719 (533 bp)	#	5	NT_34772.6 (N)	42576639-42577171 (533 bp)
	R	GCCGTGGGCGATTATGAGAATG (0, 0)		#	5	AC021965.3 (L)	94060—94592 (533 bp)
**#35**	F	ACAGCCATTCTCATCCAAACCC (0, 0)	11654—12195 (542 bp)	▴	5	NW_001838952.2 (N)	4525902—4526443 (542 bp)
	R	GGTCGTAAGCCTCTGTTGTCAG (0, 0)		▴	5	AC008670.6 (L)	83113—83654 (542 bp)
**#36**	F	GCTCACTCACCCACCACAT (0, 0)	12009—12462 (454 bp)	—	—	—	—
	R	GGATGCGACAATGGATTTTA (0, 0)					
**#37**	F	ACCACCCTAACCCTGACTTCC (0, 0)	12358—12848 (491bp)	—	—	—	—
	R	GCTTGAATGGCTGCTGTGTTG (0, 0)					
**#38**	F	GATGATACGCCCGAGCAGATG (2, 1)	12806—13311 (506 bp)	•	5	NW_001838952.2 (N)	4527054—4527559 (506 bp)
	R	TGCTAGGTGTGGTTGGTTGATG (2, 1)		•	5	AC008670.6 (L)	84265—84770 (506 bp)
**#39**	F	TCCACTTCAAGTCAACTAGGAC (0, 0)	13249—13785 (537 bp)	—	—	—	—
	R	GGGGATTGTTGTTTGGAAGGG (0, 0)					
**#40**	F	GCAGCCGGAAGCCTATTCG (0, 0)	13708—14070 (363 bp)	—	—	—	—
	R	TGAGGTGATGATGGAGGTGGAG (0, 0)					
**#41**	F	ATCACACACCGCACAATCCC (0, 0)	13930—14371 (442 bp)	—	—	—	—
	R	ATTGGTGCTGTGGGTGAAAGAG (0, 0)					
**#42**	F	TCCTCCCGAATCAACCCTGAC (0, 0)	14261—14706 (446 bp)	—	—	—	—
	R	TCATTGGTCGTGGTTGTAGTCC (1, 2)		#	5	NT_34772.6 (N)	7695705—7696149 (445 bp)
**#43**	F	AATAACACACCCGACCACAC (0, 0)	14548—14992 (445 bp)	#	5	AC021965.3 (L)	97423—97867 (445 bp)
	R	AAGGTAGCGGATGATTCAGC (2, 1)					
**#44**	F	TCATCAATCGCCCACATCACTC (0, 0)	14936—15341 (406 bp)	—	—	—	—
	R	ATAGGAGGTGGAGTGCTGCTAG (0, 0)					
**#45**	F	AGACAGTCCCACCCTCACAC (0, 0)	15256—15743 (488 bp)	—	—	—	—
	R	GGAGGTCTGCGGCTAGGAG (0, 0)					
**#46**	F	CTCCGATCCGTCCCTAACAAAC (0, 0)	15587—16185 (599 bp)	—	—	—	—
	R	GGTTTTGATGTGGATTGGGT (0, 0)					
**#47**	F	ACATTACTGCCAGCCACCATG (0, 0)	16098—16456 (359 bp)	—	—	—	—
	R	CCGGAGCGAGGAGAGTAGC (0, 0)					
**#48**	F	CAGTCAAATCCCTTCTCGTCCC (0, 0)	16344—276 (502 bp)	—	—	—	—
	R	TCTGTGTGGAAAGTGGCTGTG (0, 0)					

The amplicon numbers (Amp. #) and primer sequences were obtained from the paper of Meierhofer et al [7]. F and R represent forward primer and reverse primer of each amplicon, respectively. Hits on NCBI and Hits on BioEdit indicate the number of hits with perfect match from the primer sequences with nDNA sequences in the GenBank database of the NCBI and the local database created in BioEdit, respectively, according to the BLAST search results. The possibility of 2 primers for each amplicon matching with nDNA on the same chromosome was evaluated, and the matched accession numbers, positions on the chromosome, and the sizes of the putative NUMTs are indicated. Match for NUMT location refers to the conditions of matching both primers to the same chromosome, with the following symbols indicating the matching conditions of the primers to nDNA: •, perfect match for both primers to the accession number indicated; △, perfect match for one primer and one mismatch for another primer; ▴, one mismatch for both primers; ▽, perfect match for one primer and 2 or more mismatches for another primer; ▾, one mismatch for one primer and 2 or more mismatches for another primer; #, 2 or more mismatches for both primers. N and L indicate results from NCBI and local database searches, respectively. The symbol of — indicates that no matching result for the same chromosome could be found. Although some primers could match the same chromosome, even with 2 or more mismatches, the matched primers could be positioned too far away from each other to practically generate PCR products. In such cases, the matching results are not listed.

**Table 2:** Results for the bioinformatics analysis on the matching of SB primers to NUMTs.

Amp. #	Primer sequences and the number of perfect matches with nDNA (Hits on NCBI, Hits on BioEdit)		Amplified mtDNA region (size)	NUMT location for the primer pair that matches to the same chromosome			
				Match	Chr. #	Accession #	Position (size)
**#1 (A)**	F	GATCACAGGTCTATCACCCTA (0, 0)	1—2027 (2027 bp)	—	—	—	—
	R	TTGGACAACCAGCTATCACCA (22, 18)					
**#2 (B)**	F	GCACACCCGTCTATGTAGCA (4, 3)	1941—3948 (2008 bp)	▾	17	NT_024862.14 (N)	356087—358091 (2005 bp)
	R	TTCGATGTTGAAGCCTGAGAC (6, 3)		▾	17	NT_024862.13 (L)	339613—341617 (2005 bp)
				▾	17	AF227907.1 (L)	4660—6664 (2005 bp)
				▾	17	AC107940.13 (L)	96451—98455 (2005 bp)
**#3 (C)**	F	CCACACTAGCAGAGACCAAC (0, 0)	3869—5883 (2015 bp)	—	—	—	—
	R	GGCTGAGTGAAGCATTGGACT (4, 3)					
**#4 (D)**	F	GAAGCTGCTTCTTCGAATTTGC (5, 2)	5777—7667 (1891 bp)	—	—	—	—
	R	GGGCGTGATCATGAAAGGTG (0, 0)					
**#5 (E)**	F	CAAGTAGGTCTACAAGACGCT (4, 3)	7601—9627 (2027 bp)	•	1	NT_004350.19 (N)	46784—48808 (2025 bp)
	R	CTGATGCGAGTAATACGGATG (1, 2)		•	1	AF134583.1 (L)	3688—5712 (2025 bp)
				△	5	NT_034772.6 (N)	7701074—7703100 (2027 bp)
				△	5	AC021965.3 (L)	91360—93386 (2027 bp)
				•	6	AL359496.30 (L)	90471—92497 (2027 bp)
**#6 (F)**	F	TACCACTCCAGCCTAGCCC (2, 4)	9510—11593 (2084 bp)	△	5	AC021965.3 (L)	92381—94466 (2086 bp)
	R	TCGTAGGCAGATGGAGCTTG (2, 1)		▽		NT_034772.6 (N)	
**#7 (G)**	F	CGGCTATGGTATAATACGCCT (2, 1)	11476—13581 (2106 bp)	▽	5	AC008670.6 (L)	82935—85039 (2106 bp)
	R	AGCGATGAGAGTAATAGATAGG (2, 0)		▽	5	NT_034772.6 (N)	42572871—42574867 (1997 bp)
**#8 (H)**	F	CCTCACAGGTTTCTACTCCAA (1, 1)	13491—15493 (2003 bp)	▽	5	AC008670.6 (L)	84950—86946 (1997 bp)
	R	GAGGTCTGGTGAGAATAGTGT (0, 0)					
**#9 (H’)**	F	GCAGCCCTAGCAACACTCC (0, 0)	15314—90 (1346 bp)	—	—	—	—
	R	CAATGCTATCGCGTGCATACC (0, 0)					
**#10 (I)**	F	GAACACACAATAGCTAAGACCC (8, 2)	1045—3079 (2035 bp)	▾	8	NT_167187.1 (N)	20727551—20729527 (1977 bp)
	R	CGGTCTGAACTCAGATCACGTA (3, 1)		▾	8	NT_007995.8 (L)	190554—192530 (1977 bp)
				▾	X	AL158819.14 (L)	2580—4588 (2007 bp)
**#11 (J)**	F	CGATGTTGGATCAGGACATCC (17, 11)	2988—5061 (2073 bp)	—	—	—	—
	R	GGTTGTACGGTAGAACTGCTA (0, 1)					
**#12 (K)**	F	CATAGCAGGCAGTTGAGGTG (0, 0)	4955—7048 (2094 bp)	▽	1	NT_004350.19 (N)	44137—46231 (2095 bp)
	R	GATAGGACATAGTGGAAGTGG (2, 3)		▽	1	NW_001838563.2 (N)	2027—4120 (2095 bp)
				▽	1	AF134583.1 (L)	1042—3135 (2095 bp)
				▽	6	AL359496.30 (L)	88711—90807 (2097 bp)
**#13 (L)**	F	CTCATCACTAGACATCGTACTA (3, 4)	6983—9027 (2045 bp)	•	1	NT_004350.19 (N)	46166—48208 (2043 bp)
	R	GCCTGCAGTAATGTTAGCGG (1, 2)		▽	1	NW_001838563.2 (N)	50-2092 (2043 bp)
				•	1	AF134583.1 (L)	3070—5112 (2043 bp)
				•	6	AL359496.30 (L)	90742—92786 (2045 bp)
**#14 (M)**	F	CATCAGCCTACTCATTCAACC (4, 3)	8964—10740 (1777 bp)	—	—	—	—
	R	GTACGTAGTCTAGGCCATATG (1, 2)					
**#15 (N)**	F	GCCTAGCCCTACTAGTCTCAA (2, 3)	10690—12769 (2080 bp)	▾	5	NT_034772.6 (N)	7697929—7700008 (2080 bp)
	R	CTCAGCCGATGAACAGTTGG (0, 0)		▾	5	AC021965.3 (L)	93563—95642 (2080 bp)
**#16 (O)**	F	CGTTACATGGTCCATCATAGAA (0, 0)	12621—14700 (2080 bp)	—	—	—	—
	R	GTCGTGGTTGTAGTCCGTGC (1, 2)					
**#17 (P)**	F	CTCCTCAATAGCCATCGCTG (2, 1)	14462—1045 (3193 bp)	—	—	—	—
	R	GGGTCTTAGCTATTGTGTGTTC (5, 2)					

The 17 SB amplicons were designated as A to P amplicons by Bannwarth et al., but we have reassigned it to SB#1 to SB#17 for better illustration. Amp. # represents amplicon #. Primer sequences were obtained from the paper of Bannwarth et al. [11]. F and R represent forward primer and reverse primer of each amplicon, respectively. Hits on NCBI and Hits on BioEdit indicate the number of hits with perfect match from the primer sequences with nDNA sequences in the GenBank database of the NCBI and the local database created in BioEdit, respectively, according to the BLAST search results. The possibility of 2 primers for each amplicon matching with nDNA on the same chromosome was evaluated, and the matched accession numbers, positions on the chromosome, and the sizes of the putative NUMTs are indicated. Match for NUMT location refers to the conditions of matching both primers to the same chromosome, with the following symbols indicating the matching conditions of the primers to nDNA: •, perfect match for both primers to the accession number indicated; △, perfect match for one primer and one mismatch for another primer; ▽, perfect match for one primer and 2 or more mismatches for another primer; ▾, one mismatch for one primer and 2 or more mismatches for another primer. N and L indicate results from NCBI and local database searches, respectively. The symbol of — indicates that no matching result for the same chromosome could be found. Although some primers could match the same chromosome, even with 2 or more mismatches, the matched primers could be positioned too far away from each other to practically generate PCR products. In such cases, the matching results are not listed.
