# Supplementary figures and images for: Interference of Co-Amplified Nuclear Mitochondrial DNA Sequences on the Determination of Human mtDNA Heteroplasmy by Using the SURVEYOR Nuclease and the WAVE HS System
Source: PLoS One. 2014 Mar 24;9(3):e92817. doi: 10.1371/journal.pone.0092817 (PMC3963942; doi:10.1371/journal.pone.0092817)

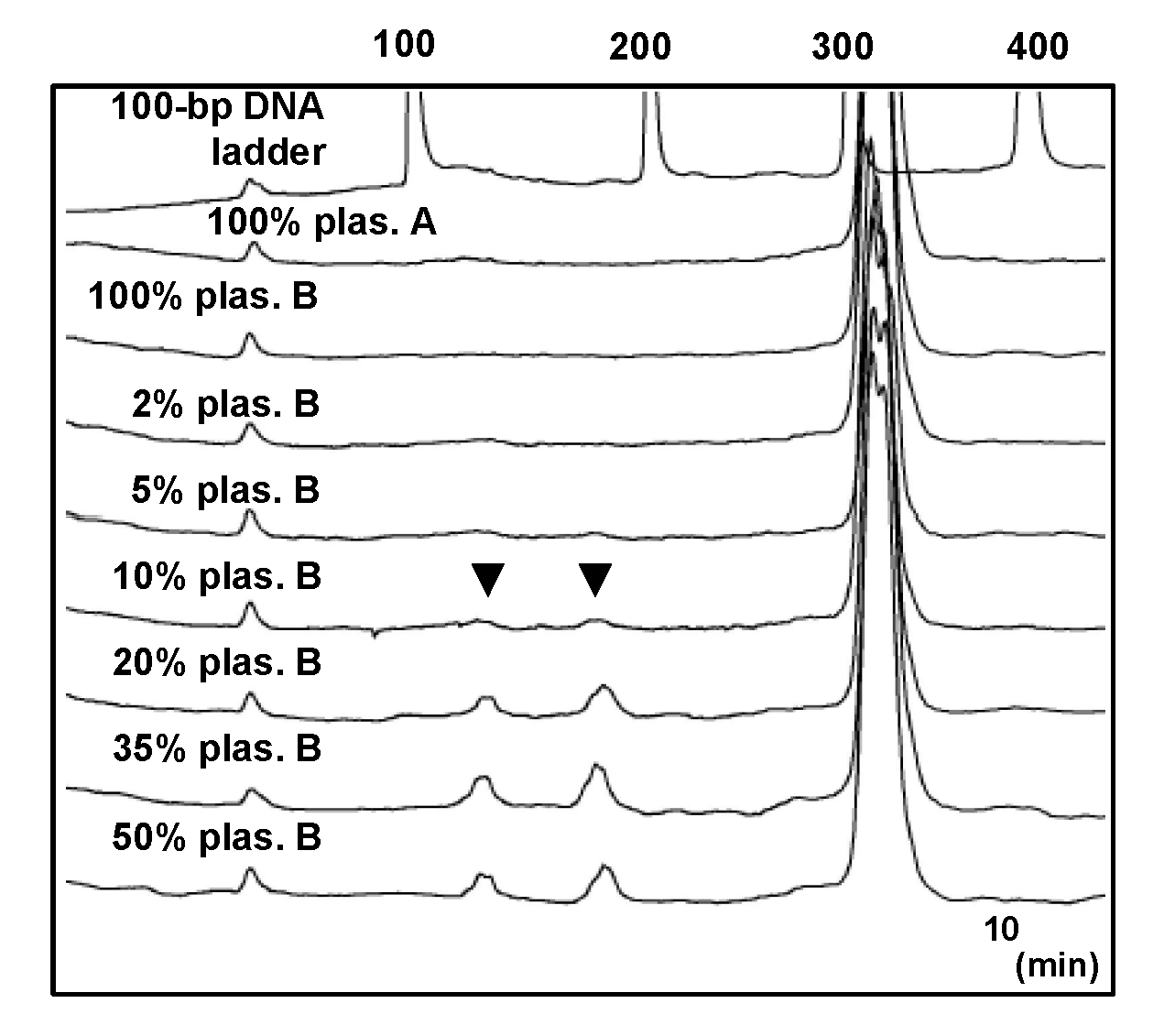

Supplement: Figure S1 — Simultaneous detection of the same DNA samples used in Figure 2B by using the UV detector during SN/WAVE-HS analysis. During the detection of the DNA by the HS detector on the WAVE System, the data acquired by the UV detector of the WAVE System were recorded simultaneously. ▾ indicates the expected DNA fragments in the sizes of 139 bp and 190 bp after SN digestion of heat-annealed PCR products. (TIFF) [file pone.0092817.s001.tiff]

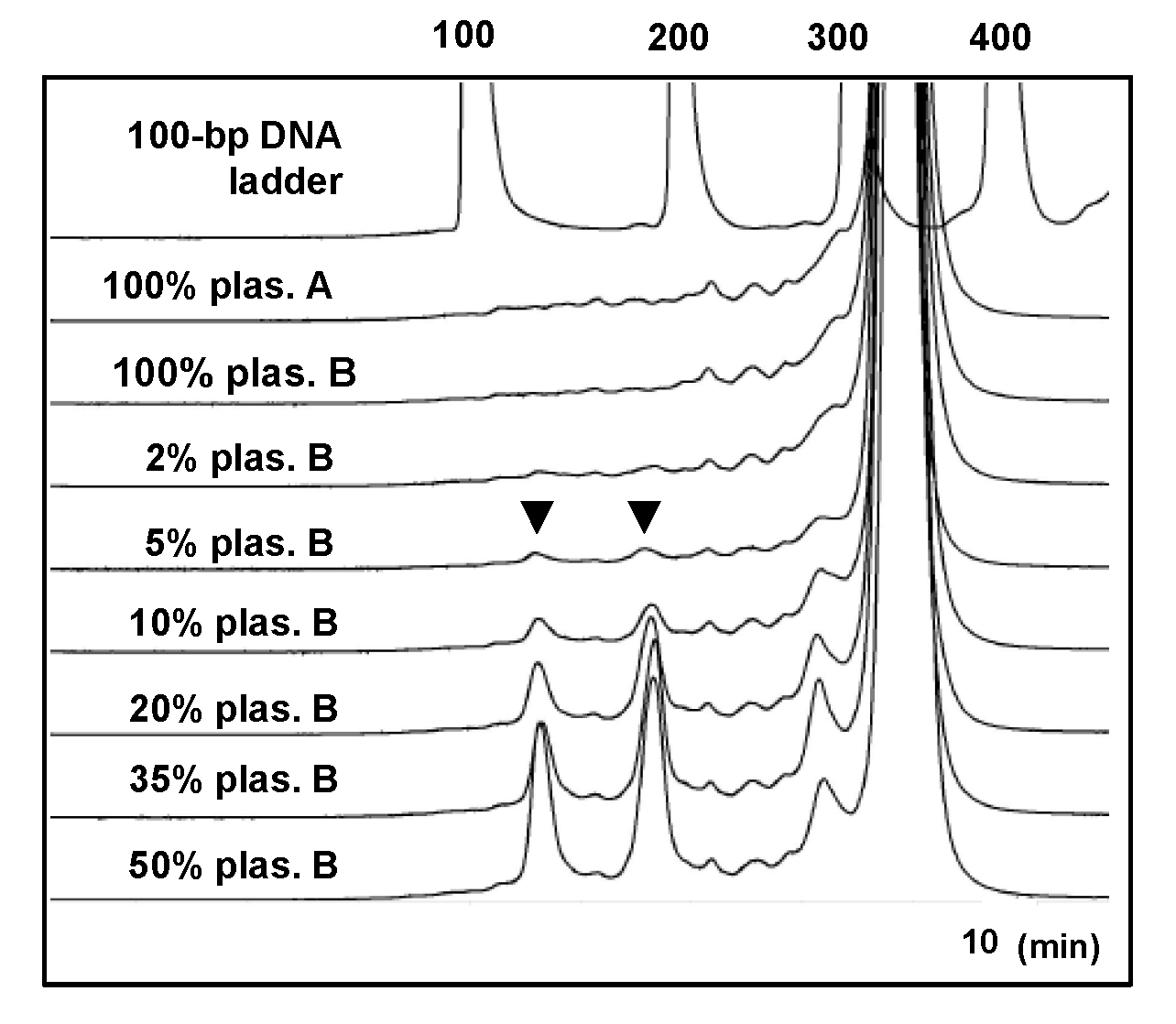

Supplement: Figure S2 — SN/WAVE-HS analysis of the same PCR products used in Figure 2 by using the old SN kit. The same heat-annealed PCR products were analyzed by SN/WAVE-HS by using the SURVEYOR Mutation Detection Kit. (TIF) [file pone.0092817.s002.tif]
