# Supplementary material for: Interference of Co-Amplified Nuclear Mitochondrial DNA Sequences on the Determination of Human mtDNA Heteroplasmy by Using the SURVEYOR Nuclease and the WAVE HS System
Source: PLoS One. 2014 Mar 24;9(3):e92817. doi: 10.1371/journal.pone.0092817 (PMC3963942; doi:10.1371/journal.pone.0092817)
Supplement: Table S1 — Positions in the DM#38 amplicon (nt 12806–13311) with different nucleotide sequences among 143B-ρ0 cells, 143B cells, and the cybrid cells. The mtDNA sequences between 143B and the cybrid cells are only different at position 13135, which is indicated by the bold and italic fonts. (PDF) [file pone.0092817.s003.pdf]

**Table S1. Positions in the DM#38 amplicon (nt 12806—13311) with different nucleotide sequences among 143B-$\rho^0$ cells, 143B cells, and the cybrid cells.**

| **Position** | **143B-$\rho^0$ cells** | **143B** | **Cybrid** |
|---|---|---|---|
| 12982 | T | C | C |
| 13011 | T | C | C |
| 13020 | C | T | T |
| 13023 | T | C | C |
| 13062 | T | A | A |
| 13105 | G | A | A |
| 13111 | C | T | T |
| ***13135*** | A | ***G*** | ***A*** |
| 13140 | A/G | A | A |
| 13145 | A | G | G |
| 13164 | C | A | A |
| 13174 | C | T | T |
| 13242 | G | A | A |
| 13260 | C | T | T |
| 13272 | T | C | C |
| 13281 | C | T | T |

The mtDNA sequences between 143B and the cybrid cells are only different at position 13135, which is indicated by the bold and italic fonts.
