# Supplementary material for: Interference of Co-Amplified Nuclear Mitochondrial DNA Sequences on the Determination of Human mtDNA Heteroplasmy by Using the SURVEYOR Nuclease and the WAVE HS System
Source: PLoS One. 2014 Mar 24;9(3):e92817. doi: 10.1371/journal.pone.0092817 (PMC3963942; doi:10.1371/journal.pone.0092817)
Supplement: Table S2 — Positions in the SB#12 amplicon (nt 4955–7048) with different nucleotide sequences among 143B-ρ0 cells, 143B cells, and the cybrid cells. The mtDNA sequences between 143B and the cybrid cells are different at several positions, which are indicated by the bold and italic fonts. (PDF) [file pone.0092817.s004.pdf]

Table S2. Positions in the SB#12 amplicon (nt 4955—7048) with different nucleotide sequences among 143B-ρ^0 cells, 143B cells, and the cybrid cells.

Position	143B-ρ^0 cells	143B	Cybrid
4960	C	***G***	***C***
4961	A	***G***	***A***
4991	A	G	G
5041	C	T	T
5147	A	G	G
5320	T	C	C
5351	G	A	A
5387	T	C	C
5426	C	T	T
5471	A	G	G
5474	G	A	A
5498	G	A	A
5580	C	T	T
5821	A	G	G
5840	T	C	C
6023	A	G	G
6221	C	***C***	***T***
6242	T	C	C
6266	C	A	A
6267	G	***A***	***G***
6299	G	A	A
6366	A	G	G
6371	C	***T***	***C***
6383	T	C	C
6410	T	C	C
6452	T	C	C
6483	T	C	C
6512	C	T	T
6542	T	C	C
6569	A	C	C
6641	C	T	T
6935	T	C	C
6938	T	C	C

7028	C	***T***	***C***
7039	T	***A***	***T***

The mtDNA sequences between 143B and the cybrid cells are different at several positions, which are indicated by the bold and italic fonts.
